# Selective Deuteration of Neopentyl Glycol and Its Effect on the Plastic Crystal Transformation

**DOI:** 10.1021/acs.jpcb.5c08152

**Published:** 2026-01-30

**Authors:** Chase B. Somodi, Vanaparthi Satheesh, Tzu-Hsuan Chao, Daniel P. Tabor, Emily B. Pentzer, Patrick J. Shamberger

**Affiliations:** † Department of Materials Science and Engineering, 14736 Texas A&M University, College Station, Texas 77843, United States; ‡ Department of Chemistry, 14736 Texas A&M University, College Station, Texas 77843, United States

## Abstract

Transitions between rotationally ordered and disordered states in globular small molecules are associated with large entropy changes and thus hold promise as solid-state barocaloric refrigerants. However, the relationships between elements of the molecular structure and the corresponding thermodynamic properties of the phase transformation between ordered and disordered states remain unresolved. We hypothesize that more spherical molecules, as measured by their rotational moments of inertia, exhibit larger relative increases in their rotational degrees of freedom as they transition to rotationally disordered states. We probe this by isolating the impact of rotational moments of inertia from more dominant factors, including intermolecular bonding, through the selective deuteration of different functional groups of the model plastic crystal molecule neopentyl glycol. We demonstrate a decrease in the phase transition temperature of up to approximately 3 K associated with the existence of deuterated methyl groups and explain this change in terms of relative changes in the rotational moments of inertia of the compounds. This observation places bounds on the role of rotational moments of inertia in thermodynamic aspects of the phase transformation and introduces a vector for subtle modulation of the transition point for cooling applications.

## Graphical abstract

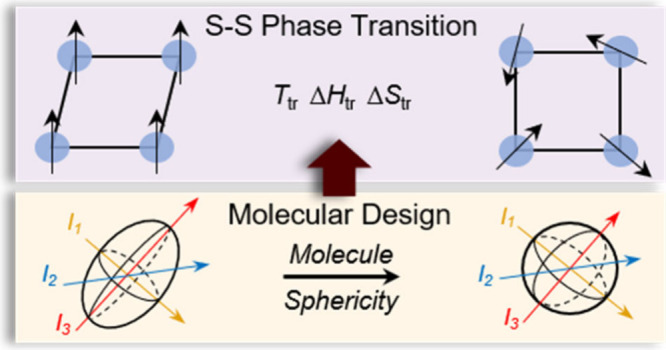

## 1 Introduction

The atmospheric abundance of the hydrofluorocarbon (HFC) refrigerant R-134A increased over 233,000% in the 15-year span of 1990–2005,^1^ and its continued use is predicted to contribute to up to 0.45 K of global temperature rise by the year 2100.^2^ In contrast, solid–solid caloric refrigeration schemes offer a unique alternative to vapor compression systems as they are inherently leak-free, in most cases are nontoxic, and can exhibit high efficiencies.^3^ These materials rely on solid–solid caloric effects to store and release heat and are driven by changes in external fields like magnetic, electric, and stress.^4,5^ Among this class of materials, barocaloric effects have been exhibited in both organic^6–12^ and inorganic^13,14^ materials and can be associated with high magnitudes of isothermal entropy and adiabatic temperature changes.

Recent studies have discovered a colossal barocaloric effect in plastic crystals, including the molecule studied herein, neopentyl glycol (NPG).^12^ Despite this promise, questions remain regarding the fundamentals of plastic crystals, most notably the properties that impact thermodynamic quantities of plastic crystal phase transitions. In many plastic crystal compounds, like neopentyl glycol, intermolecular hydrogen bonds favor the formation of ordered crystal phases, and the breaking of these hydrogen bonds allows the molecules to acquire rotational freedom, forming the plastic crystal phase. Thus, these bonds play a significant role in the thermodynamics of the phase transition. The role of the shape of the molecule in determining the thermodynamics of the phase transition, as determined by the relative molecular rotational moments of inertia, is not as well understood. In this study, we aim to isolate and evaluate the impacts of molecular shape through selective deuteration to understand its contributions to the thermodynamic characteristics of NPG. Success in this goal could provide a blueprint for control over the thermodynamics of plastic crystals, potentially helping to engineer them for refrigeration, heat pumps, and thermal energy control applications. Furthermore, success could also give insights into the impact of torsional modes on the thermodynamics of solid–solid phase transitions, particularly rotational order–disorder transformations.

Barocaloric effects have been observed for a number of solid–solid phase transitions described by different mechanisms, including spin crossover transitions,^15–19^ chain melting transitions,^14,20–22^ and rotational order–disorder transitions.^6,7,12^ Molecular solids which undergo a phase transition from a rotationally ordered low symmetry crystal structure to a high symmetry rotationally disordered crystal structure are classified as “rotator phases” or also as “plastic crystals”.^23,24^ This latter name arises from the remarkably deformable nature of the high symmetry rotationally disordered state relative to its low symmetry rotationally ordered state.^23,25^ This increased deformable nature is due in large part to the number of facile slip planes in the cubic crystal structure plastic crystals typically adopt at higher temperature.^26,27^ Most often, plastic crystals are identified in small, globular organic molecules, as these systems provide the necessary framework to be able to (i) lock their orientation at low temperature and (ii) perform dynamic reorientations once a sufficient thermal energy is achieved to weaken the intermolecular bonds that hinders rotation.^28–30^ The transition from rotational order to disorder produces an entropy change that is large relative to the total entropy change associated with a transition from a crystalline solid to a liquid state (with both translational and rotational degrees of freedom).^31^ The combination of a transition possessing a large entropy change that can be driven by both temperature and pressure motivates plastic crystals as promising candidates for various thermal energy storage, heat pumping, and refrigeration applications. 2,2-Dimethyl-1,3-propanediol, commonly referred to as neopentyl glycol (NPG) has been studied extensively for this reason, as it exhibits an isothermal entropy change of 510 J·kg^–1^·K^–1^ at applied pressures of 0.57 GPa.^12^

Intermolecular hydrogen bonding has been observed to play a dominant role in the determination of the thermodynamic properties of plastic crystal phase transitions. In the case of NPG, hydrogen bonding impacts the transition between rotational order and disorder. At low temperature, intermolecular hydrogen bonds lock the orientation of NPG molecules, while at high temperature such intermolecular hydrogen bonds are broken, allowing for orientational disorder.^7,32^ Doping NPG with Li-ion salts disrupted the hydrogen bonding network in NPG, leading to a disappearance of the plastic crystal phase transition above 9.09 mol % lithium salt.^33^ Additionally, previous studies showed that systematically increasing the number of hydroxymethyl groups on the neopentane skeleton, and therefore the number of intermolecular hydrogen bonds per molecule, increased the enthalpy, entropy and temperatures of transition.^34–36^ These findings suggest that polar groups capable of hydrogen bonding play a major role in formation of the plastic crystal phase, thus having a profound impact on the thermodynamic properties of these organic molecular crystals.^36^ This effect has also been observed in primary amine-containing plastic crystals where increases in the number of polar amine groups similarly led to increases in the enthalpy, entropy and temperatures of transition.^37^ Notably, hydrogen bonding is not a requirement for an orientationally disordered phase to exist as molecules such as fullerene^38^ (C_60_) and tungsten hexafluoride (WF_6_) exhibit an orientationally disordered phase before melting.^39^

Molecular shape is understood to play a crucial role in whether a plastic crystal phase will be exhibited by an organic molecule, but its role in the determination of the thermophysical properties is not well understood. Fundamentally, the globular shape of molecules provides the necessary framework to allow for molecular reorientation, and thus orientational disorder within a crystalline lattice.^23,28,30^ Therefore, deviation from sphericity may reasonably be anticipated to affect the entropy of the plastic crystal transition, as asymmetrically shaped molecules could reasonably be expected to affect the equilibrium ensemble of rotational states and thus the entropy change associated with relaxing to this ensemble of rotationally disordered states. Isolating the impacts of molecular shape on thermodynamic properties is difficult, as incorporating different functional groups alters both the shape and the intermolecular interactions. Kobashi et al.^40^ investigated the role of molecular sphericity in the thermodynamics of a set of molecules and concluded that deviation from sphericity of the molecule gives rise to positional and/or orientational short-range order, which consequently could be responsible for observed decreases in the entropy of fusion. An alternative strategy to analyze the role of sphericity is to alter a molecule’s moment of inertia while conserving intermolecular forces utilizing selective deuteration.

In previous studies, selective deuteration proved to be an effective strategy in the study of solid–solid phase transitions, particularly for probing the nature of phase transitions and the impacts of the geometric isotope effect on the thermodynamics of rotational order–disorder transitions. Selective deuteration of norbornane showed an increase in the temperature of the solid–solid phase transition by upward of 25 K with increased deuteration count. This change in transition temperature was postulated to result from changes to the moment of inertia, and thus the shape of the molecule.^41^ However, increasing the number of selectively deuterated functional groups does not always result in increases in the temperature of a solid–solid phase transition. A study on the geometric isotope effect for the solid–solid phase transition in anthracene-tetracyanobenzene showed that increasing the number of deuterated functional groups lowered the transition temperature.^42^ Similar observations of a transition temperature decrease with increased deuteration were made in a hydrogen-bonded host–guest crystal. Remarkably, this study also showed that deuteration altered the donor–acceptor distance in O–H···O hydrogen bonds, resulting in the emergence of additional phases not observed in the nondeuterated compound.^43^ Given the importance of the hydrogen bond network for the plastic crystal transition in NPG, selective deuteration studies could broaden the understanding of the role of both hydrogen bonding through donor–acceptor distance modulation while also examining the role of molecular shape and sphericity in plastic crystal transitions. Kamae and co-workers^36^ reported two deuterated analogues of NPG, focusing on the impacts of deuteration on the low temperature solid–solid phase transition at approximately 60 K, leaving unevaluated the plastic crystal transition.

Here, we examine NPG and seven selectively deuterated analogues by focusing our attention on the effect of deuteration on the thermodynamic aspects of the plastic crystal transition. We selectively deuterate the hydroxy groups (−OH), methylene groups (−CH_2_−), and methyl groups (−CH_3_) both as individuals and in combinations (Figure 1). Calorimetry was used to investigate and analyze the impact of composition on the temperature, enthalpy change, entropy change, and heat capacity of the compounds. These results are compared with molecular shape metrics determined via conformer sampling, providing insights into the role of molecular shape on the plastic crystal phase transitions. Experimental analysis reveals that selective deuteration of the methyl group (NPG-d6) results in a subtle decrease of up to 3 K in the plastic crystal transition temperature. Shape analysis was performed using computational analysis and showed that the plastic crystal transition temperature is correlated with the change in moment of inertia associated with transitioning from the rotationally ordered to disordered phase. These results both constrain the magnitude of changes associated with shape effects in this particular transition and introduce a new vector for shifting transition temperatures of plastic crystals.

**Figure 1:**
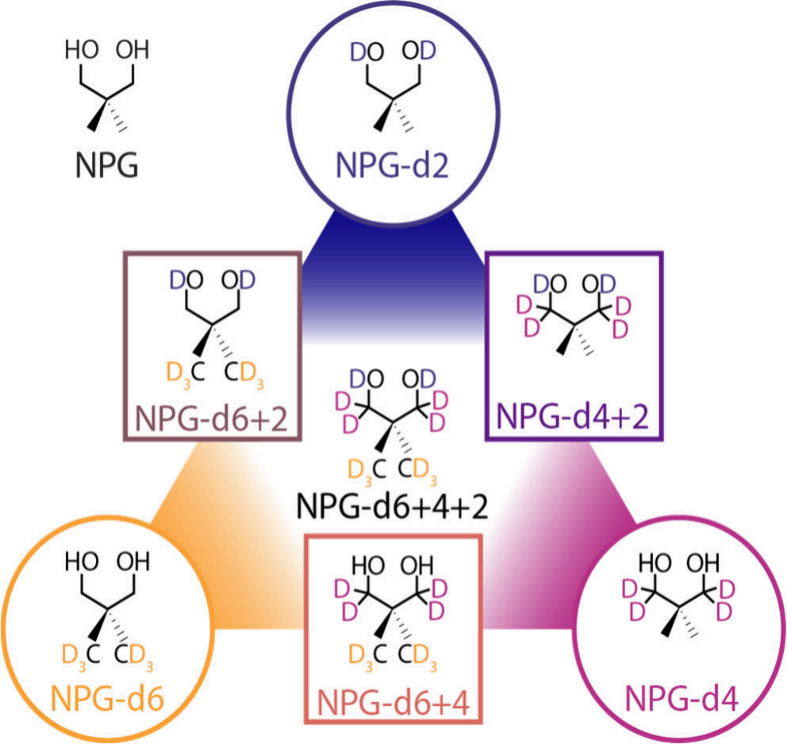
Selective deuteration of NPG to produce the molecules of study: deuteration of the hydroxy group (NPG-d2), the methylene group (NPG-d4), the methyl group (NPG-d6), dual deuterations (NPG- d4+2, NPG- d6+2, and NPG-d6+4) as well as the fully deuterated compound (NPG-d6+4+2).

## 2 Methods

### 2.1 Materials

#### 2.1.1 Reagents

Starting reagents used in this study include NPG (99%, Alfa Aesar); diethyl malonate (99%), lithium aluminum hydride (LAH 95%), and sodium hydride (NaH) in 60% mineral oil (Sigma-Aldrich); diethyl dimethyl malonate (97%) and iodomethane-d3 (99+%, Acros); lithium aluminum deuteride (LAD 98%, Strem Chemicals); diethyl ether (ACS reagent), tetrahydrofuran (THF, HPLC grade), sodium chloride (ACS grade), and Celite (Fischer Scientific); magnesium sulfate (MgSO_4_, 99%) and sodium hydroxide (NaOH 98%, Oakwood Chemicals); and deuterium oxide (D_2_O 99.9%, Cambridge Isotopes). NPG was purified via sublimation at 323.15 K prior to *n*-pentane and dichloromethane (DCM) solvent wash. Remaining chemicals were used as received.

#### 2.1.2 Synthesis

Selective deuteration of neopentyl glycol was performed through a two-step process involving methylation, followed by reduction. Synthesis began with the methylation of diethyl malonate using deuterated methyl iodide (CD_3_I) to install the methyl (CD_3_) groups (-d6 position). Subsequently, diethyl 2,2-dimethylmalonate-d6 was reduced using lithium aluminum deuteride (LiAlD_4_) to install deuterated methylene (CD_2_) groups (-d4 position). Deuterium exchange was then performed using heavy water to deuterate the hydroxyl groups (-d2 position). Combinations of these methods were used to produce the remaining selectively deuterated samples, described in detail for each compound in Supporting Information Section 2. A trace impurity was detected (^1^H NMR, <0.5%) at a chemical shift of approximately 4 ppm in all derivates of NPG, including the commercially available NPG. This impurity led to significant inconsistencies in measured thermodynamic quantities, as outlined in the Supporting Information, Section 5, emphasizing the importance of chemical purification in analysis of these compounds. For appropriate purification, all compounds were subjected to sublimation at 323.15 K prior to being washed with *n*-pentane and methylene chloride.

### 2.2 Characterization

#### 2.2.1 Chemical and Elemental Analysis

^1^H and ^13^C nuclear magnetic resonance (NMR) spectroscopy was performed to evaluate purity and monitor reaction on a Bruker Avance NEO 400 MHz (Figure 2); chloform-3 (99%) was used as the solvent for all samples, with the residual chloroform peak used as reference (7.2 ppm). Gas chromatography mass spectrometry (GCMS) was performed by the TAMU Mass Spec facility to confirm predicted molecule mass and check for impurities. Lastly, elemental analysis was performed by Atlantic Microlab.

**Figure 2:**
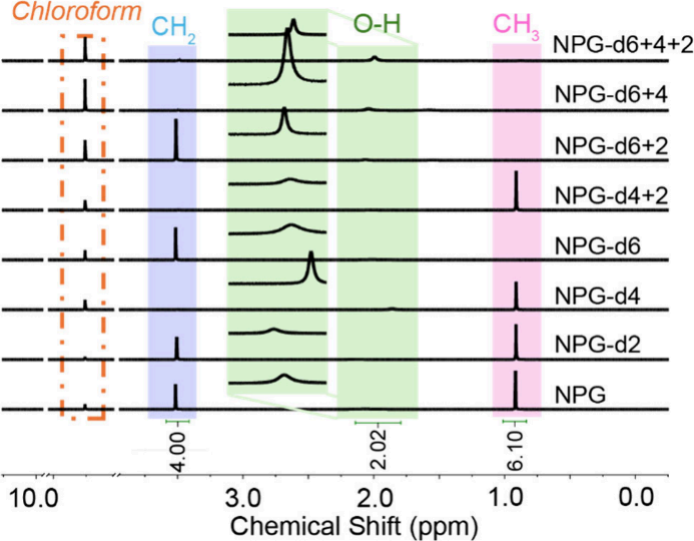
^1^H NMR spectra of as received NPG and all deuterated analogues. The blue, green, and pink areas highlight the peaks corresponding to the methylene (−CH_2_−), hydroxyl (−OH), and methyl (−CH_3_) groups, respectively. The inset green area at the chemical shift amplifies the signal attributed to −OH (approximately 2.0 ppm) for the sake of clarity.

#### 2.2.2 Calorimetry

Thermal analysis of NPG and the selectively deuterated analogues was carried out using two differential scanning calorimeters (DSC). The transition temperatures, enthalpy changes, and entropy changes were determined using a TA Instruments Q2000 DSC. For this method, samples of approximately 5 to 10 mg of the powdered forms of each compound were prepared in aluminum DSC pans and hermetically sealed. Prior to data collection, each sample was heated to 413.15 K in order to (i) erase the prior thermal history and (ii) form a contiguous volume in good thermal contact with the bottom of the DSC pan. Following this premelt, samples were equilibrated and held at 273.15 K for 5 min followed by heating to 413.15 K at a scanning rate of 2 K·min^–1^. Repeated measurement of indium standards provided predicted relative uncertainties of *u*_r_(*T*_fus_) = ±0.0004 and *u*_r_(Δ*H*_fus_) = ±0.025 at 95% confidence interval, respectively. Calibration of the device was confirmed by analyzing the deviations between the measured and reference values for the standard performed on the Q2000, which were within ±0.0003 and ±0.02 for *T*_*fus*_ and Δ*H*_*fus*_, respectively.

To measure isobaric heat capacities, a Setaram Microcalvet Microcalorimeter was used. Approximately 30 to 40 mg of NPG were prepared in sealed vessels (Hastelloy C276). These samples were melted under a dry N_2_ atmosphere to prevent contamination by moisture. Samples were initially equilibrated at 243.15 K for 20 min, scanned in a continuous heating mode at 0.2 K·min^–1^, and then held isothermal at 393.15 K. Repeated measurement of deionized water provided a predicted relative uncertainty of *u*_r_(*C*_p,m_) = ±0.015 at a 95% confidence interval over the temperature range of interest. To confirm the calibration of the measurement, we repeatedly measured and analyzed the molar heat capacity of hexadecane. Comparing the molar heat capacities obtained via measurement to references at various temperatures provided relative deviations within ±0.02 for *C*_p,m_.

### 2.3 Simulation

#### 2.3.1 Conformational Space Analysis

The conformational space of neopentyl glycol was explored using the conformer-rotamer sampling tool (CREST) search protocol, which employs the semiempirical GFN2-xTB method to efficiently sample low-energy conformers within a 6 kcal/mol window from the global minimum. A total of 20 conformers were identified by this procedure. Removing conformations with intramolecular hydrogen bonding reduced the number of conformers sampled to 15. All remaining conformations represent local minima in the conformational landscape for an individual molecule. These intramolecular torsional minima will be similar in the condensed phase. These structures were subsequently reoptimized at the ωB97X-D/def2-TZVPP level of theory using Gaussian­[G16] to obtain more accurate geometries. Normalized principal moments of inertia (NPRs) were then calculated for each optimized conformer. To determine the NPRs, the moment of inertia tensor was first computed and diagonalized to extract its three principal components. These components sorted in ascending order are denoted as *I*_1_, *I*_2_ and *I*_3_. The shape descriptors were then calculated using the expressions npr1 = *I*_1_/*I*_3_ and npr2 = *I*_2_/*I*_3_.

#### 2.3.2 Molecular Dynamics Simulations

Molecular dynamics simulations of the ordered crystal phase were performed with GROMACS.^44^ The initial cell parameters and atomic structures were obtained from the Cambridge Crystallographic Data Centre (CCDC).^45^ Force field parameters were generated using ACPYPE^46–48^ employing the AMBER force field. A time step of 1 fs was used throughout the simulations. The initial configuration was constructed by replicating the experimental unit cell into a 10 × 5 × 5 supercell containing 1000 molecules in total. The corresponding supercell parameters were *a* = 59.82800 Å, *b* = 53.62500 Å, *c* = 50.21346 Å, α = 90°, β = 99.7747°, and γ = 90°. The system was first energy minimized for 500 steps, followed by a 1 ns equilibration under the *NVT* ensemble at a fixed temperature of 200 K by using a velocity-rescale thermostat. Subsequently, a 20 ns production run was carried out under the *NPT* ensemble with a Parrinello–Rahman barostat at 1 bar. Configurations from the final 10 ns were used for analysis as the potential energy during the initial 10 ns indicated that the system had reached equilibrium. The final cell parameters were as follows: *a* = 59.43060 Å, *b* = 53.58920 Å, *c* = 50.10959 Å, α = 90.0000°, β = 99.7952°, and γ = 90.0000°.

## 3 Results and Discussion

### 3.1 Thermodynamic Properties of the Phase Transition

Differential scanning calorimetry (DSC) analysis reveals that the plastic crystal transition temperature decreases by up to 3 K for molecules in which the methyl group (-d6 position) of NPG is deuterated, in comparison to nondeuterated analogues. The thermodynamic properties for the plastic crystal transition in all tested compounds are listed in Table 1. The endothermic peaks at lower temperature correspond to the plastic crystal solid–solid (S–S) phase transition. For molecules in which the methyl groups are deuterated, the onset and peak temperature shift to lower values than the nondeuterated analogues (Table 1; Figure 3). For the molecules NPG-d6, NPG-d6+2, NPG-d6+4, and NPG-d6+4+2, the average onset temperature *T*_tr_ is 312.3 K, while all other compounds have an average *T*_tr_ of 315.1 K. In contrast, there is no discernible impact on the enthalpy change or entropy change associated with the plastic crystal transition within the accuracy of the measurements. These observations are consistent with previous reports for NPG, NPG-d2 and NPG-d6+4+2.^36^ A shoulder is observed near the measured onset temperature for NPG-d6+4+2 which leads to larger uncertainty; this may be the result of trace contaminant that is not detected (e.g., not observed in ^1^H NMR or GC). The endothermic peak for all compounds at higher temperature corresponds to melting, or the solid–liquid (S–L) transition. Contrary to the solid–solid (S–S) plastic crystal transition, calorimetric analysis of fusion data does not show the same dependence of S–L transition on deuteration of the methyl group (Table S2 in the Supporting Information). To elucidate the origin of the downward shift in the temperature of the S–S plastic crystal transition observed with a deuterated methyl group (-d6), the molar mass of each compound was compared to the data for the phase transition obtained via calorimetry (Figure 4). The temperature of transition shows the same differences between molecules with and without the -d6 deuteration: no clear separation or dependence is discernible for the enthalpy and entropy of the phase transition. However, this approach to the question only accounts for the static mass of the molecule, whereas the transition involves the activation of molecular vibrational modes and full molecular rotation, motivating further the evaluation of the change in moment of inertia.

**Table 1:** Thermodynamic Properties of the Plastic Crystal Transition in Deuterated NPG

	*T*_*tr*_ ^a^ (K)	Δ*H*_*tr*_ ^b^ (J·g^–1^)	Δ*H*_*tr*_ ^b^ (kJ·mol^–1^)	Δ*S*_*tr*_ ^b^ (J·mol^–1^·K^–1^)
NPG	314.8	119 ± 4	12.4 ± 0.4	39.4 ± 1.4
NPG-d2	314.8	116 ± 2	12.3 ± 0.2	39.2 ± 0.7
NPG-d4	315.3	120 ± 1	13.0 ± 0.1	41.0 ± 0.3
NPG-d4+2	315.6	119 ± 2	13.1 ± 0.3	41.6 ± 0.8
NPG-d6	312.2	114 ± 6	12.5 ± 0.7	40.1 ± 2.2
NPG-d6+2	312.2	110 ± 2	12.3 ± 0.4	39.4 ± 1.1
NPG-d6+4	312.8	111 ± 2	12.7 ± 0.2	40.6 ± 0.6
NPG-d6+4+2	312.1	108 ± 17	12.6 ± 2.0	40 ± 6

a *u*_r_(*T*) = ±0.0004 and the uncertainty is ±0.1 K for all molecules.

b *u*_r_(Δ*H*_tr_) = ±0.025. The reported uncertainties for Δ*H*_tr_ and Δ*S*_tr_ are ±2σ of the number *n* individually prepared samples, where *n* = 2 for NPG-d6+4+2, and 3 for all other compounds.

**Figure 3:**
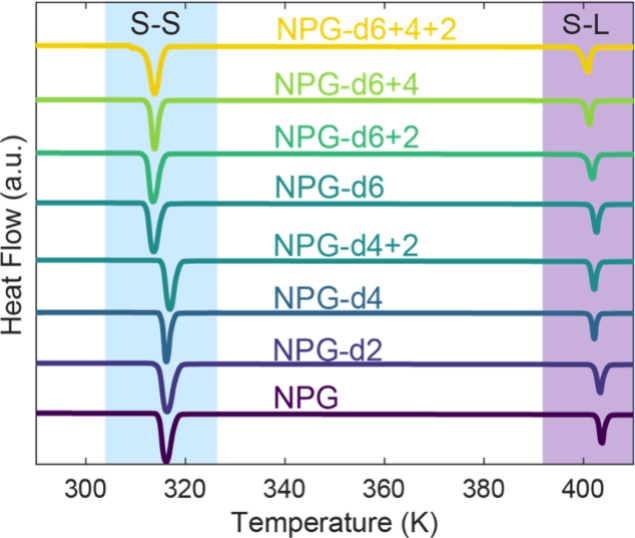
Endothermic transformations of NPG and its selectively deuterated analogues determined via differential scanning calorimetry (DSC). The plastic crystal transition (S–S) and melting transition (S–L) are highlighted in blue and purple, respectively. All samples were prepared in hermetically sealed aluminum DSC pans and scanned at 2 K·min^–1^ following an initial premelt at 413 K. Signals are offset for sake of visualization.

**Figure 4:**
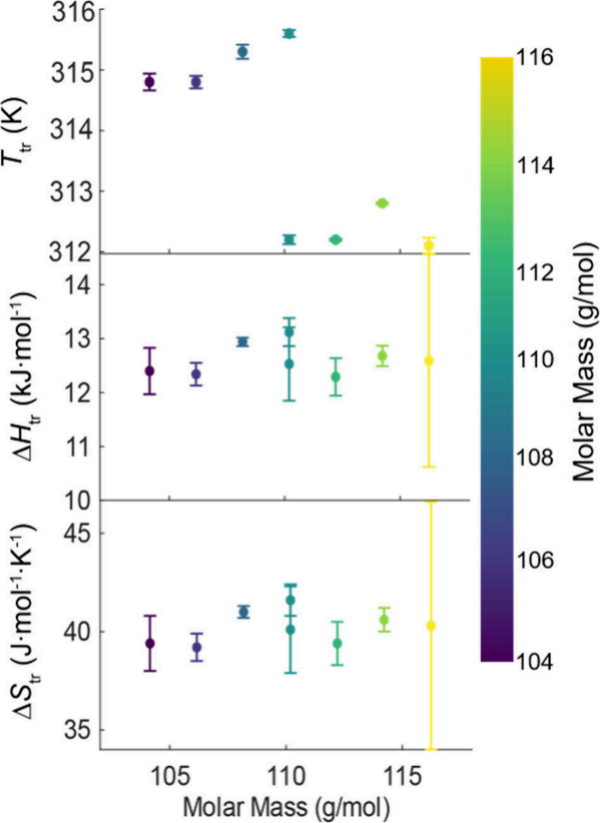
Thermodynamic properties of the plastic crystal (S–S) transition and their dependence on the molar mass. The error bars are ±2σ of the number *n* individually prepared samples, where *n* = 2 for NPG-d6+4+2 and 3 for all other compounds.

### 3.2 Impact of Selective Deuteration on the Moments of Inertia

#### 3.2.1 Conformational Analysis

Selectively substituting hydrogen with the heavier deuterium isotope imparts changes in the distribution of mass within a molecule. It has been well established that the plastic crystal transition in NPG involves a change in crystal structure from monoclinic to FCC, accompanied by the onset of rotation of the molecules about their center of mass within the FCC lattice and associated increase in rotational degrees of freedom.^7,12^ A recent investigation has revealed that in addition to this onset of full molecular rotation, the hydroxymethyl (−CH_2_OH) group also begins to rotate about the C–C bond axis during the plastic crystal transition.^49^ This discovery implies that within one molecule type, the family of accessible conformations in the rotationally disordered states and, thus, the relative changes in rotational degrees of freedom at the S–S transition will likely differ with different deuteration configurations. Thus, understanding how the molecular mass distribution (i.e., moment of inertia) changes during the transition within each molecule of study and how these changes correlate to the thermodynamic properties is the focus of this analysis. The 15 lowest energy unique conformers determined via the CREST conformer search protocol for a single molecule in a vacuum provide the conformational landscape that each molecule of study could adopt in their condensed phase (Figure 5b). Utilizing molecular dynamics simulations and crystallographic constraints, conformations could be assigned as either ordered or plastic crystal conformations. Combining this analysis with experimental data from calorimetry could help pinpoint the potential origins of the bimodal dependence of the transition temperature, as outlined in Section 3.1.

**Figure 5:**
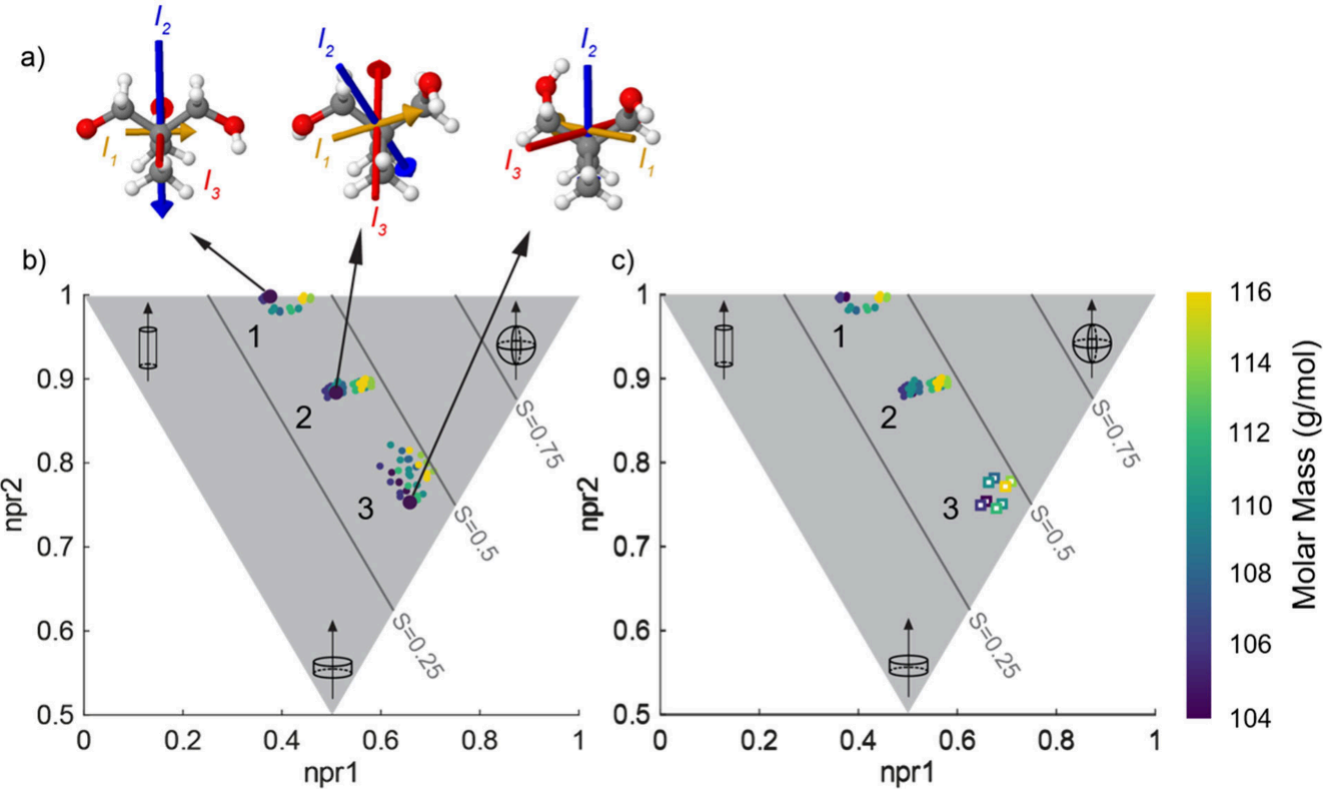
a) Representative molecular conformations of NPG across the three clusters, each associated with different orientations of the hydroxymethyl (−CH_2_–OH) functional groups. Normalized principal moment of inertia (npr) space for b) the 15 lowest energy conformations of each molecule determined via the CREST conformer search protocol and c) the conformations of the ordered crystal phase confirmed from crystallographic data (squares) and unique conformations accessible in the plastic crystal phase (circles). In both b) and c), labels 1 and 2 correspond to plastic crystal conformational clusters, which correspond to different orientations of the C–C bond on the hydroxymethyl functional group, as shown in a), while label 3 corresponds to the ordered crystal conformations. Solid gray lines in b) and c) represent the sphericity contours defined by S = npr_1_ + npr_2_ – 1, shown for values of S = 0.25, 0.50, and 0.75.

#### 3.2.2 Ordered Crystal Phase Conformations

To assess the accessible conformations in the ordered crystal phase of each deuterated molecule, molecular dynamics simulations were used in conjunction with energy constraints from the monoclinic phase of NPG. Prior results suggest that molecules in the ordered crystal phase adopt specific constrained orientations of the hydroxymethyl groups (−CH_2_–OH) to form intermolecular hydrogen bonds and that the molecules primarily rest in a singular conformation and orientation.^7,33,50,51^ Here, molecular dynamics simulations provided sample conformations of the ordered crystal phase in the temperature range below the plastic crystal transition for each molecule of study (Figure S32 in the Supporting Information). Comparing these conformations determined via MD to the 15 lowest energy conformers determined via the CREST conformer search protocol, it was determined that cluster 3 alone (Figure 5b) represents the ordered crystal phase. Notably, these conformations exhibit the orientation of the hydroxymethyl group capable of forming the intermolecular hydroxy bond that locks the molecules in place, as illustrated for the representative conformer from this cluster (rightmost representation in Figure 5a). To confirm the validity of the determination that cluster 3 represents the ordered crystal phase, the conformation of standard, nondeuterated NPG molecules observed in the monoclinic ordered crystal phase were determined from crystallographic data obtained previously.^45^ The hydrogens of this crystallographic conformation of NPG were then substituted for deuterium, providing a single representative ordered crystal phase conformation for each molecule (squares, Figure 5c). These conformations fell within the range sampled from both MD simulations and the CREST conformer search protocol and exemplified the orientation of the hydroxymethyl group capable of forming intermolecular hydroxy bonds. Clusters 1 and 2, as determined via CREST, were notably absent from both molecular dynamics simulations and crystallographic data recorded below the plastic crystal transition temperature. Thus, the conformations that match cluster 3 determined from prior crystallographic data were used as the representative ordered crystal phase conformations in the final analysis.

#### 3.2.3 Plastic Crystal Phase Conformations

The plastic crystal phase of NPG features molecules occupying the lattice sites of an FCC phase, rotating about their center of mass. MD simulation of conformations exhibited in the plastic crystal phase is challenging due to the coexistence of long-range translational order and dynamically disordered molecular rotations. Though MD simulations were attempted, they did not adequately reproduce the cubic crystal structure of the plastic crystal phase. However, at elevated temperature in the liquid regime, conformations consistent with all three groups are observed, supporting the claim that these clusters are representative of low-energy conformations in condensed phases (Figure S33 in the Supporting Information). The rotationally dynamic nature of NPG, including the rotation of the hydroxymethyl group, points to a family of accessible conformations coexisting at any point in time in the plastic crystal phase. Thus, we hypothesize that clusters 1 and 2 represent the range of new conformations that are accessible upon transition to the plastic crystal phase, but not in the ordered crystal phase. Importantly, conformations from cluster 3 could be represented in the plastic crystal phase, but these have been neglected as the interest here is the change in conformational shape between the rotationally ordered and disordered phases, and the associated changes in rotational degrees of freedom. For the sampled conformer in cluster 1, the hydroxymethyl groups are nearly symmetric about the principal moment *I*_2_ (left most representation, Figure 5a). Comparing this to the sampled conformer in cluster 2 (middle representation, Figure 5a), it is evident that one of the hydroxymethyl groups has rotated, consequently altering the magnitude and direction of the principal axes. We anticipate that this rotation of the hydroxymethyl group prohibits the formation of intermolecular hydroxy bonds, as demonstrated in the ordered crystal sampled conformer (right most representation, Figure 5a). This could further confirm the hypothesis that these conformers represent a family of unique orientations found only after transitioning to the plastic crystal phase. Lastly, for both ordered and plastic crystal conformers, deuterated molecules tend to be more spherical, particularly for molecules in which the methyl group is deuterated (Figure 5a,b). The average sphericity, calculated as S = npr1 + npr2 – 1, for molecules with and without methyl group deuteration in the ordered crystal phase (cluster 3, Figure 5c) is 0.45 and 0.43, respectively.

### 3.3 Impact of Moment of Inertia on the Thermodynamic Properties of the Plastic Crystal Transition

The temperature of the plastic crystal transition is related to the overall sphericity of the molecule (Figure 5), implying a relationship between the distribution of mass within the molecules and the change in rotational degrees of freedom associated with the rotational order–disorder S–S transition. To further decompose the relative changes in moment of inertia associated with the S–S transition, we calculated the difference between the normalized principal moments of inertia (npr1 and npr2) of newly accessible conformations within the plastic crystal phase, (cluster 1 and 2) and the ordered crystal phase (cluster 3). We interpret the difference between normalized principal moments of inertia as a proxy for changes in rotational degrees of freedom (i.e., Δ*S*_rot_) associated with the order–disorder transition. Given the large experimental uncertainty in the enthalpy and thus the calculation of entropy, this difference was instead compared against the temperature of the S–S transition in each molecule determined via calorimetry (Figure 6). Given that each cluster consists of multiple data points, a Boltzmann-weighted probability was calculated using the relative energies of each cluster. Because the ordered crystal phase is assigned to one conformation (Section 3.2.2), the probability used in this calculation corresponds to the relative probabilities of conformations in clusters 1 and 2 independently. The cumulative results suggest that a strong correlation exists between the change in the first normalized principal moment of inertia (Δnpr1) and the temperature of the plastic crystal transition (Figure 6a,c). Conversely, the scattered data for the difference in the second normalized principal moment of inertia (Δnpr2) for both clusters provide a weak correlation (Figure 6b,d). Notably, these results do not significantly change when a different conformation from the ordered crystal conformer is selected, as they are nearly equidistant to clusters 1 and 2 and similar in overall sphericity. This dependence of transition temperature on the difference in npr1 but not npr2, as well as the bimodal dependence of the transition temperature on whether the methyl group is deuterated or not, can be understood by examining how mass is distributed in the ordered and plastic crystal phases.

**Figure 6:**
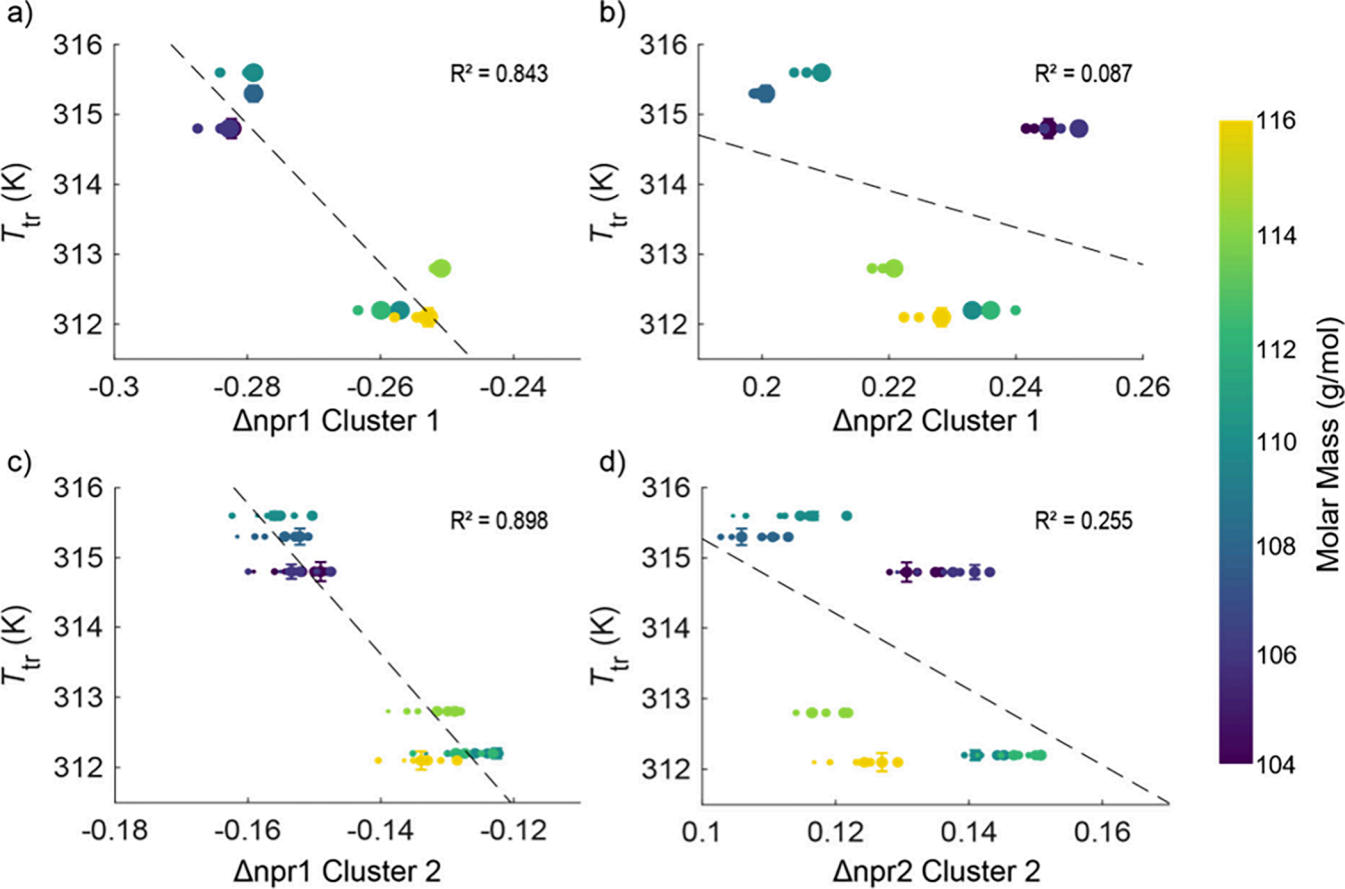
Dependence of transition temperature on the change in a) the first normalized principal moment of inertia (npr1) and b) the second (npr2) going from cluster 3 (ordered crystal) to cluster 1 (plastic crystal). The same calculation for the change in c) npr1 and d) npr2 going from cluster 3 to cluster 2. The diameters of the markers correspond to the Boltzmann-weighted probability determined by the relative energies within each respective cluster. The representative error bar is centered on the single most probable conformation within each cluster and represents ±2σ of the number *n* individually prepared samples, where *n* = 2 for NPG-d6+4+2 and 3 for all other compounds. The dashed lines indicate the weighted linear regression.

The dependence of the transition temperature on the difference in npr1 but not npr2 can be understood by observing how the principal moments are oriented throughout the transition. In the ordered crystal conformer (rightmost representation of Figure 5a) and the conformer sampled from the first of two clusters corresponding to plastic crystal phase conformations (leftmost representation of Figure 5a), the second principal moment (*I*_2_) is observed to roughly bisect the methyl and hydroxymethyl groups. This signifies that mass is largely symmetric about this principal axis with the only significant change being the reorientation of the hydroxymethyl group. Conversely, the first and third principal moments (*I*_1_ and *I*_3_) are oriented such that mass is asymmetrically distributed about the plane that these moments form (Figure S34 in the Supporting Information). In the sampled conformer from the second cluster corresponding to the plastic phase (middle representation of Figure 5a), the orientation of *I*_2_ is such that it is less symmetric. However, the orientations of both *I*_1_ and *I*_3_ have also reoriented (relative to the ordered conformer), and mass is still asymmetrically distributed about the plane that these moments form. It is this elevated degree of asymmetry of mass distribution in the first and third moments relative to the second moment and their relation to the first normalized principal moment (npr1 = *I*_1_/*I*_3_) that leads to a correlation of the plastic crystal transition temperature to the difference in the first normalized principal moment of inertia. Finally, it is both (i) the location relative to the plane formed by the principal moments *I*_1_ and *I*_3_ and (ii) the number of hydrogens associated with the methyl group, which results in the bimodal dependence of transition temperature on whether or not the methyl group is deuterated. We interpret the observations of a smaller difference in npr1 upon transitioning to a rotationally disordered state, as well as the heightened average measure of sphericity for molecules with methyl group deuteration, as being indicative of a larger overall change in rotational degrees of freedom (i.e., Δ*S*_rot_) compared to molecules without deuteration of the methyl group. This consequently results in a decrease in the plastic crystal transition temperature, as demonstrated via calorimetry.

## 4 Conclusions

Here, we have systematically investigated the impact of selective deuteration on the thermodynamics of the plastic crystal transition in the model plastic crystal neopentyl glycol (NPG). Utilizing calorimetric data from NPG and seven deuterated analogues, we demonstrate that selective deuteration induces a bimodal response, resulting in either no measurable change or a decrease in the plastic crystal transition of approximately 3 K. We observe a clear relationship between the deuteration of the methyl groups and a lowering of the plastic crystal transition temperature. We further analyzed this shift in transition temperature by coupling these experimental data with the principal moments of inertia determined via the CREST conformer search protocol. By evaluating the 15 lowest energy conformations that each molecule could adopt in the ordered and plastic crystal phases and calculating the corresponding principal moments of inertia, we analyzed the overall shift in the moment of inertia on going from the ordered to plastic crystal phase. We demonstrated a correlation between the change in the first normalized principal moment of inertia and the associated temperature of the plastic crystal transition. We interpret this correlation as being indicative of the extent of change in rotational degrees of freedom (Δ*S*_rot_) upon transitioning to a rotationally disordered state, where a larger increase in Δ*S*_rot_ results in a depression of the temperature of transition.

The observations and analysis contained herein provide new strategies to fine-tune the temperatures of the plastic crystal transition. This is promising, as heat pump and refrigeration applications often require narrow temperature windows that depend on the specific application. The ability to tune the thermodynamic properties of plastic crystals by manipulating the number of functional groups engaged in hydrogen bonding has been well-established.^34–36^ The findings of our study propose a new strategy to narrowly control the temperature of the plastic crystal transition on the molecular scale. It should be noted that other plastic crystal molecules may exhibit greater temperature shifts upon the substitution of hydrogen for deuterium. For example, the transition temperature of pentaerythritol was observed to decrease 19 K in response to 80 mol % deuteration of the hydroxy groups.^52^ Though experimental uncertainty provided challenges for evaluating the impact of shape on the enthalpy and entropy changes, we observed very small changes to both values overall, suggesting that deuteration could be a strategy to separate the desired tunable parameters. Overall, a correlation between changes in rotational moments of inertia and the transition temperature is apparent, yielding clear evidence of a change in rotational kinetic energy, confirming the dynamic rotational nature of these organic molecules.

## Supplementary Material



## Data Availability

The molecular dynamics simulation data supporting the findings of this study are available on Figshare at DOI 10.6084/m9.figshare.30670778

## Notes

General information, synthesis and purification procedure for all deuterated analogues; FT-IR, NMR, and mass spectra confirming purity of all deuterated analogues; identification of a trace impurity; microcalorimetric heat capacity analysis of neopentyl glycol; differential scanning calorimetry data for fusion thermodynamics; thermodynamic properties adjusted to a common reference temperature; molecular dynamics simulations and analysis of conformations sampled in the condensed phases; and analysis of the dependence of plastic-crystal transition temperature on molecular sphericity (PDF)
