# Supplementary material for: Selective Deuteration of Neopentyl Glycol and Its Effect on the Plastic Crystal Transformation
Source: J Phys Chem B. 2026 Jan 30;130(6):2005–14. doi: 10.1021/acs.jpcb.5c08152 (PMC12908117; doi:10.1021/acs.jpcb.5c08152)
Supplement: Supplementary file 1 [file jp5c08152_si_001.pdf]

Selective Deuteration of Neopentyl Glycol and its Effect on the Plastic Crystal Transformation

Chase B. Somodi,[1] Vanaparthi Satheesh,[2] Tzu-Hsuan Chao,[2] Daniel P. Tabor,[2] Emily B. Pentzer[1,2] and Patrick J. Shamberger[1]*

[1]Department of Materials Science and Engineering, Texas A&M University, College Station, TX, 77843, USA

[2]Department of Chemistry, Texas A&M University, College Station, TX, 77843, USA

*Denotes corresponding author

Corresponding Author Email: patrick.shamberger@tamu.edu

Table of Contents

Entry	Information	Page
1	General Information	2
2	Synthesis of Deuterated Neopentyl Glycol Derivatives	3
3	FT-IR Spectra of Compounds	5
4	NMR (^{1}H and ^{13}C) Spectra of Compounds	6
5	Detection of Trace Impurity	15
6	Mass Spectra of Compounds	16
7	Thermophysical Properties	19
8	Conformational Analysis	22
9	Sphericity Analysis	24
10	References	24

1. General Information

Starting reagents used in this study include neopentyl glycol (NPG 99%, Alfa Aesar); diethyl malonate (99%), lithium aluminum hydride (LAH 95%), and sodium hydride (NaH) in 60% mineral oil (Sigma Aldrich); diethyl dimethyl malonate (97%) and iodomethane-d3 (99+%, Acros); lithium aluminum deuteride (LAD 98%, Strem Chemicals); Diethyl ether (ACS reagent), Tetrahydrofuran (THF, HPLC grade), sodium chloride (ACS grade), and Celite (Fischer Scientific); magnesium sulfate ($MgSO_4$, 99%) and sodium hydroxide (NaOH 98%, Oakwood Chemicals); deuterium oxide (D_2O 99.9%, Cambridge Isotopes) and Chloroform-d ($CDCl_3$ 99%, Cambridge Isotopes).

Neopentyl glycol was purified under sublimation at 50 °C prior to n-pentane and DCM solvent wash, and remaining chemicals were used as received. NMR spectra were recorded on a 400 MHz Bruker spectrometer. Proton (^1H) chemical shifts are reported in parts per million (δ) relative to internally referenced to the residual proton signal of the deuterated solvent. Differential scanning calorimetry (DSC) data was collected on a Thermal Analysis Q2000 DSC.

2. Synthesis of Deuterated Neopentyl Glycol Derivatives

A) Synthetic route to NPG-d6 core derivatives:

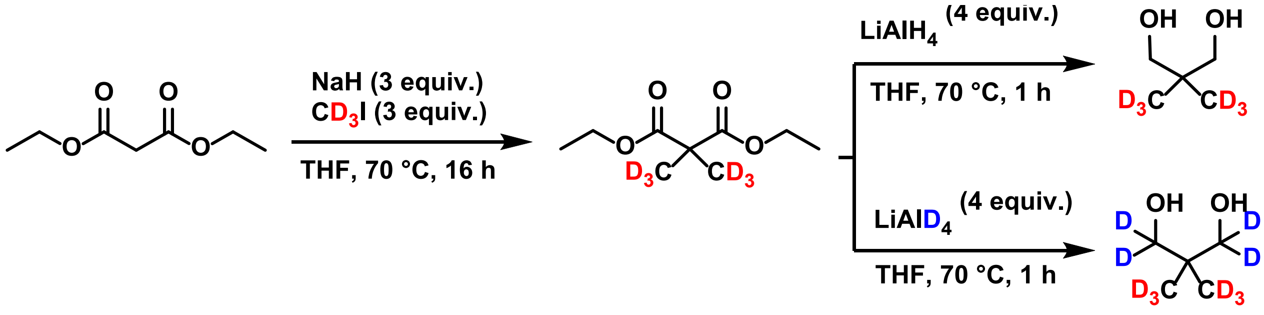

B) Synthetic route to NPG-d4 core derivatives:

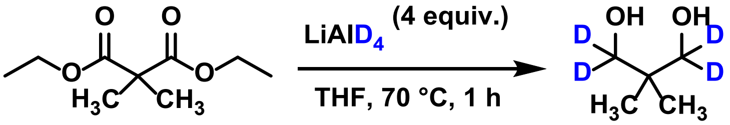

C) Synthetic route to NPG-d2 core derivatives:

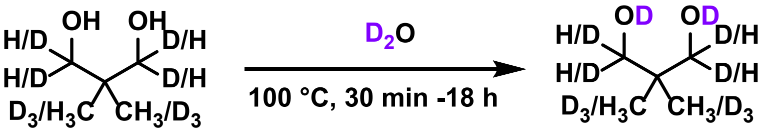

Deuterated Diethyl Dimethyl Malonate (diethyl 2,2-dimethyl-d6 malonate): Approximately 50 mL of dry THF was added to an oven dried 100 mL two-neck round bottom flask. 1.2 g (3 equiv.) of sodium hydride (NaH) was added and stirred for 15 minutes at 24 ˚C. Then diethyl malonate (1.52 mL, 1 equiv.) and idomethane-d3 (1.87 mL, 3 equiv.) were added dropwise utilizing a syringe. The solution was heated at 70 ˚C overnight while stirring. The next day the solution was quenched with deionized water via dropwise addition until the solution was clear. An extraction was performed with ethyl acetate. The combined organic layers were then washed with water and brine, then dried over $MgSO_4$ prior to vacuum filtration. The solvent was removed under reduced pressure to yield a dark yellow-brown oil.

General synthetic procedure (NPG-d4,-d6,-d6+4): Approximately 50 mL of dry THF was added to an oven-dried 100 mL two-neck round bottom flask. 4 equiv. of (about 1.52 g) lithium aluminum hydride (LAH) or lithium aluminum deuteride (LAD) was added and stirred for 15 minutes at 24 °C. Then 1.9 mL (1 equiv.) of deuterated diethyl dimethyl malonate was added dropwise via a syringe and heated to 70 °C for 1 hour. After the specified time, the reaction mixture was inserted into an ice bath and quenched it adding deionized water dropwise. THF was added to break up

the resulting metallic structure when needed. The grey liquid was then stirred for 15 minutes at 24 °C and stirred over $MgSO_4$ for an additional 15 minutes to eliminate any lithium aluminum salts. The solution was filtered over celite, and the remaining solvents were removed under reduced pressure to yield corresponding white solids. After which, the compound was subjected to sublimation at 50 °C before precipitating with an n-pentane and DCM solvent system.

NPG-d6 (1,3-Propanediol, 2,2-di(methyl-d$_3$)-): ^{1}H NMR ($CDCl_3$, 400 MHz, 298 K): δ 2.00 (s, 2H), 3.52 (s, 4H) ppm. ^{13}C NMR ($CDCl_3$, 101 MHz, 298K): δ 20.27 (m), 36.17, 71.79 ppm. HRMS-ESI$^+$ (m/z): $[M+H]^+$ calcd. For $C_5H_7D_6O_2^+$, 111.1287; found, 111.1291.

NPG-d6+4 (1,3-Propane-1,1,3,3-d4-diol, 2,2-dimethyl-d6): ^{1}H NMR ($CDCl_3$, 400 MHz, 298 K): δ 2.04 (s, 2H) and 3.48 (s, 0.15 H – 97% D) ppm. ^{13}C NMR ($CDCl_3$, 101 MHz, 298 K): δ 20.17-20.36 (m), 35.76, 70.22 (quint, *J* = 20.4 Hz) ppm. HRMS-ESI$^+$ (m/z): $[M+H]^+$ for $C_5H_3D_{10}O_2^+$ calculated, 115.1538; found, 115.1541.

NPG-d4 (1,3-Propane-1,1,3,3-d4-diol, 2,2-dimethyl-): ^{1}H NMR ($CDCl_3$, 400 MHz, 298 K): δ 0.92 (s, 6H, CH_3), 1.86 (d, 2H, OH) and 3.50 (s, 0.14 H, CH_2 – 97% D) ppm. ^{13}C NMR ($CDCl_3$, 101 MHz, 298 K): δ 21.32, 36.21, 70.90 (quint, *J* = 21.6 Hz) ppm. HRMS-ESI$^+$ (m/z): $[M+H]^+$ for $C_5H_9D_4O_2^+$ calculated, 109.1161; found, 109.1166.

General synthetic procedure (NPG-d2): 1.5 g (1equiv.) of neopentyl glycol (NPG) was dissolved in 3 mL D_2O inside a 25 mL Erlenmeyer flask. The solution was stirred while heating to 100 °C to promote the deuterium hydride exchange of the hydroxyl groups, then boiling off all present liquid while crystals formed on the sides of the vessel. This process took about 30 minutes and was repeated a second time to reach full deuteriation. After which, the NPG-d2 compound was subjected to sublimation at 50 °C prior before washing with an n-pentane and DCM solvent system.

NPG-d2 (1,3-Propanediol-d2, 2,2-dimethyl-): ^{1}H NMR ($CDCl_3$, 400 MHz, 298 K): δ 0.92 (s, 6H, CH_3), 2.15 (br s, 0.46H, CH_2 – 77% D) and 3.51 (s, 4H, CH_2) ppm. ^{13}C NMR (CDCl3, 101 MHz, 298 K): δ 21.44 (CH_3), 36.53 (quaternary C), 71.72 (CH_2) ppm. HRMS-ESI$^+$ (m/z): $[M+H]^+$ for $C_5H_{13}O_2^+$ calculated, 105.0910; found, 105.0914.

General procedure for deuterium exchange for NPG-dn+2: Other than basic NPG, deuterium exchange of remaining compounds proceeded as follows: 1.5 g of NPG-dn was dissolved in 3 mL of D_2O inside a 25 mL round bottom flask attached to a distillation apparatus with a 25 mL round bottom receiving flask. The D_2O NPG-dn solution was stirred overnight (~18 hours) at 90 °C under nitrogen atmosphere. The sample was then cooled to room temperature (10 minutes) and frozen using liquid nitrogen (10 minutes). The receiving flask was then submerged in a liquid nitrogen bath and an active vacuum was initiated for 5 minutes. After 5 mins, the vacuum was closed, and the solution mixture was heated to 35 °C. The vacuum was re-initiated when distillation was discontinued, which was indicated by disappearance of bubbling and the formation of condensate (approximately every 3 minutes). This process was repeated for another overnight heating period, followed by three consecutive days of heating. After which, the NPG-dn+2

compound was subjected to sublimation at 50 °C before washing with an n-pentane and DCM solvent system.

NPG-d6+2 (1,3-Propanediol-d2, 2,2-di(methyl-d3)-): ^{1}H NMR ($CDCl_3$, 400 MHz, 298 K): δ 3.51 (s, 4H, CH_2) and δ 2.43 (br s, 0.42 H –70% D) ppm. ^{13}C NMR ($CDCl_3$, 101 MHz, 298 K): δ 20.27–20.66 (m, CD_3), 36.11 (quaternary C), 71.73 (CH_2) ppm. HRMS-ESI$^+$ (m/z): $[M+H]^+$ for $C_5H_7D_6O_2^+$ calculated, 111.1287; found, 111.1289.

NPG-d4+2 (1,3-Propane-1,1,3,3-d4-diol-d2, 2,2-dimethyl-): ^{1}H NMR ($CDCl_3$, 400 MHz, 298 K): δ 0.91 (s, 6H, CH_3), 2.04 (br s, 0.42 H – 70% D) and 3.49 (s, 0.15H, CH_2 – 97% D), ppm. ^{13}C NMR ($CDCl_3$, 101 MHz, 298 K): δ 21.32 (CH_3), 36.03 (quaternary C), 70.79 (quint, *J* = 21.4 Hz, CD_2) ppm. HRMS-ESI$^+$ (m/z): $[M+4]^+$ for $C_5H_9D_4O_2^+$ calculated, 109.1161; found, 109.1165.

NPG-d6+4+2(1,3-Propane-1,1,3,3-d4-diol-d2, 2,2-di(methyl-d3)-)*:* ^{1}H NMR ($CDCl_3$, 400 MHz, 298 K): The internal standard indicates the purity of the compound (100% C-D_3, 99% C-D_2 and 69% O-D). ^{13}C NMR ($CDCl_3$, 101 MHz, 298 K): δ 20.52 (hept, *J* = 19.3 Hz), 35.75, 69.70 (m) ppm. HRMS-ESI$^+$ (m/z): $[M+H]^+$ for $C_5H_3D_{10}O_2^+$ calculated, 115.1538; found, 115.1538.

3. FT-IR Spectra of Compounds

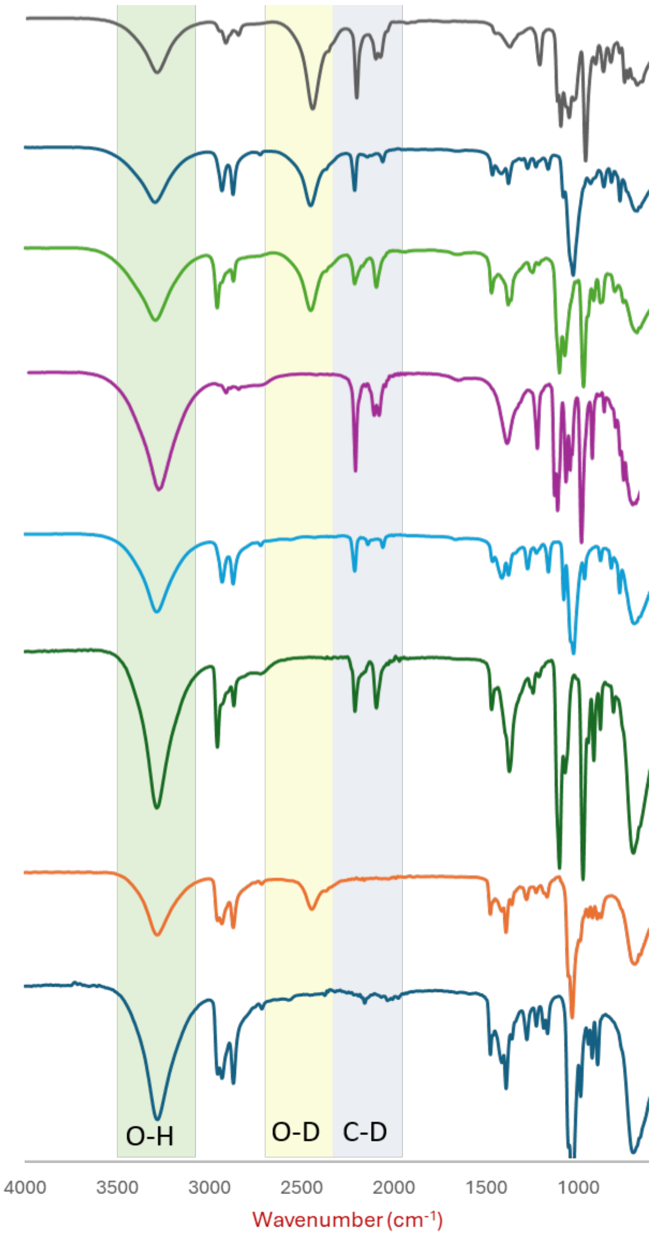

***Figure S*1.** FT-IR spectra of as received NPG and all deuterated analogs. The green, yellow, and pink areas indicate the O-H, O-D, and C-D stretching regions.

4. NMR (^1H and ^{13}C) Spectra of Compounds

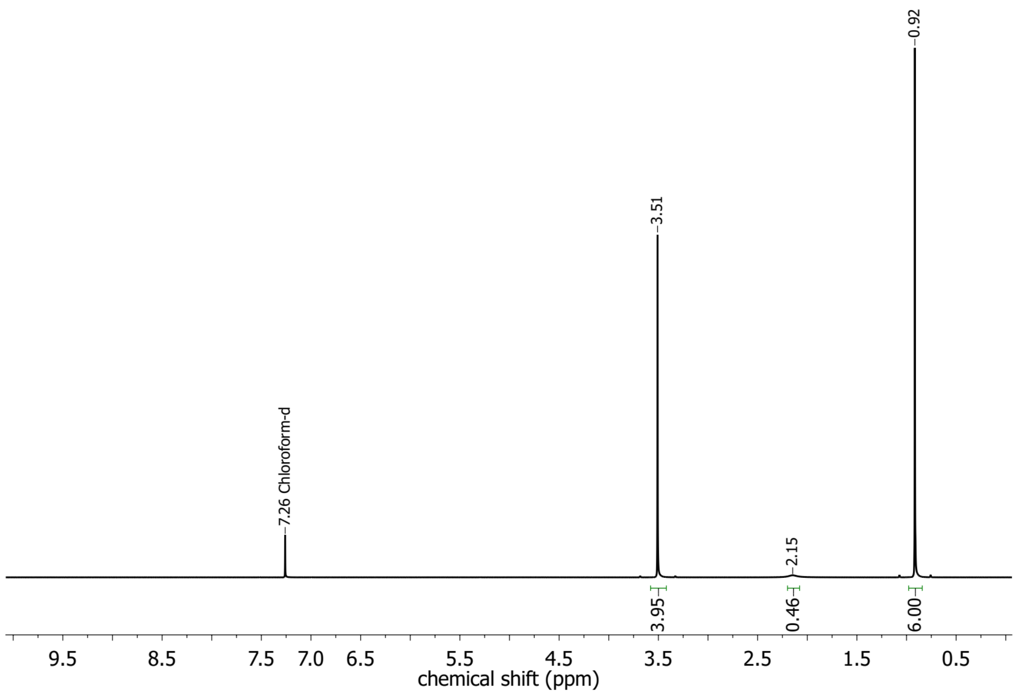

Figure S2. ^1H NMR spectrum of **NPG-d2** ($CDCl_3$, 400 MHz, 298 K)

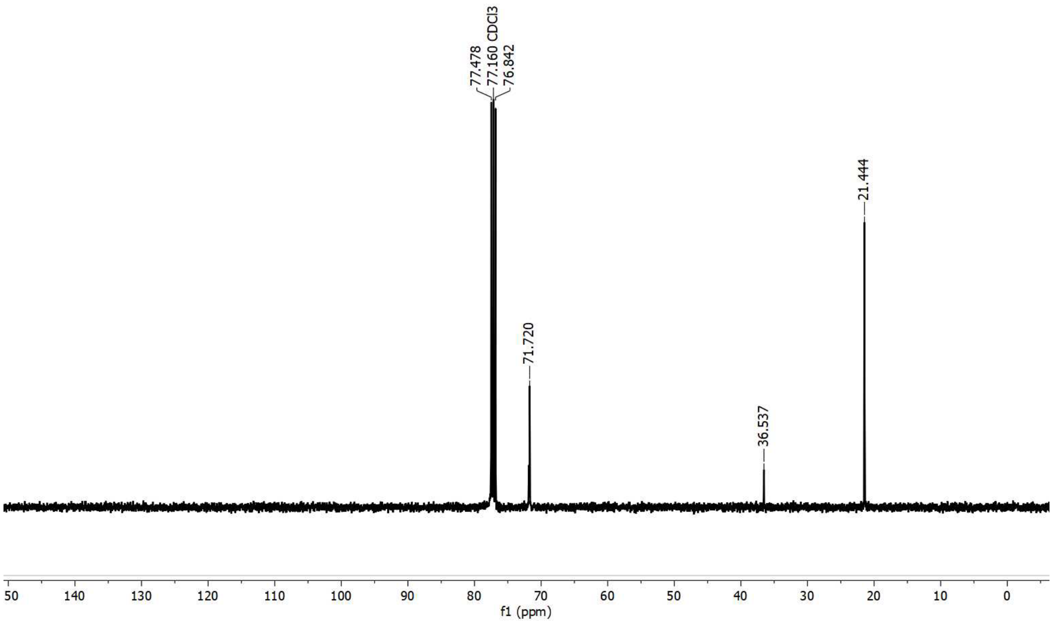

Figure S3. ^{13}C NMR spectrum of **NPG-d2** ($CDCl_3$, 400 MHz, 298 K)

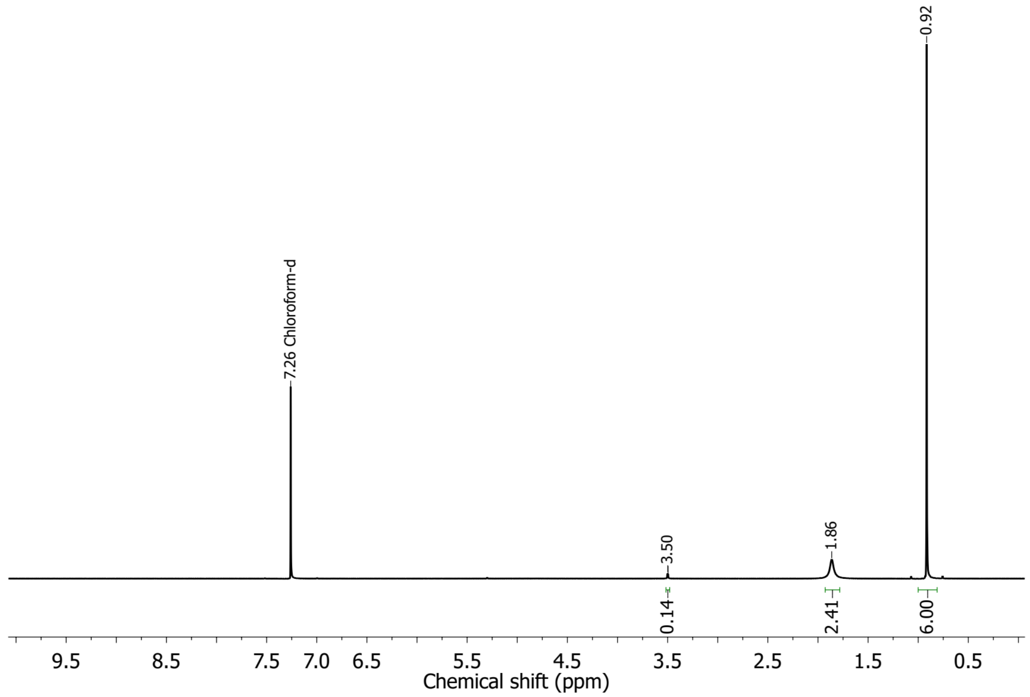

Figure S4. ^{1}H NMR spectrum of **NPG-d4** ($CDCl_3$, 400 MHz, 298 K)

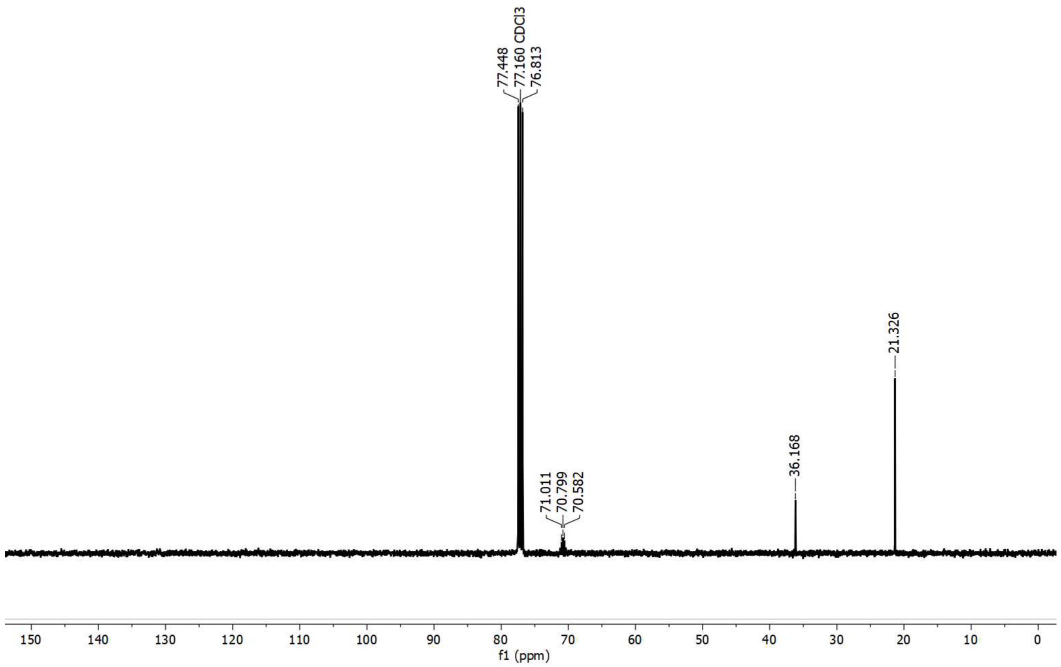

Figure S5. ^{13}C NMR spectrum of **NPG-d4** ($CDCl_3$, 400 MHz, 298 K)

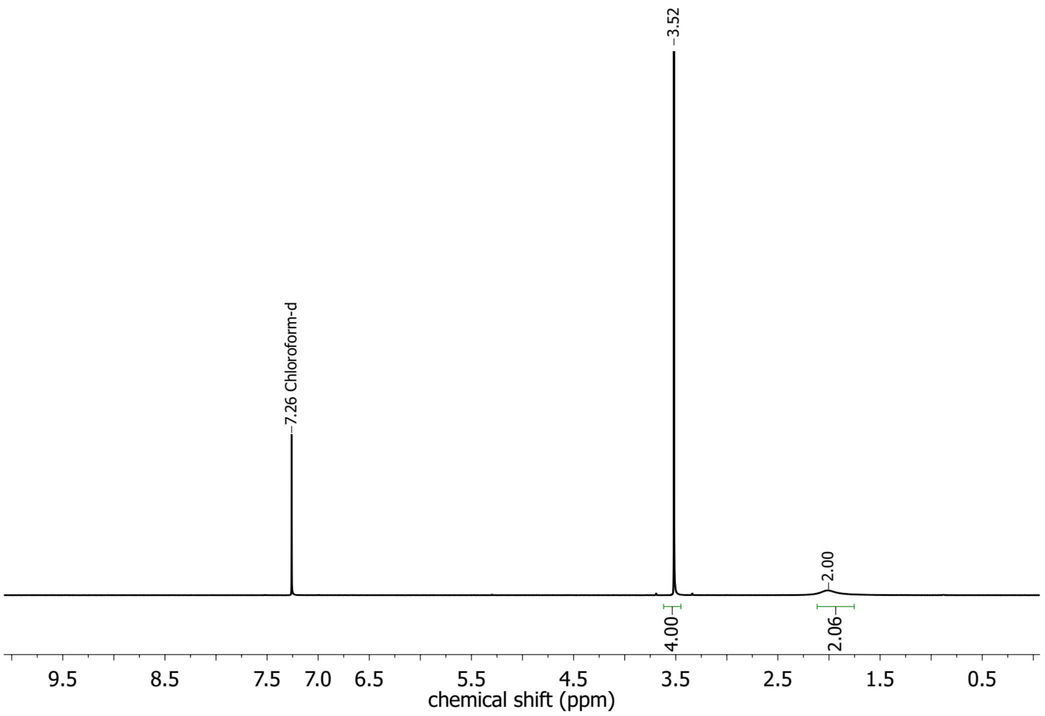

Figure S6. ^{1}H NMR spectrum of **NPG-d6** ($CDCl_3$, 400 MHz, 298 K)

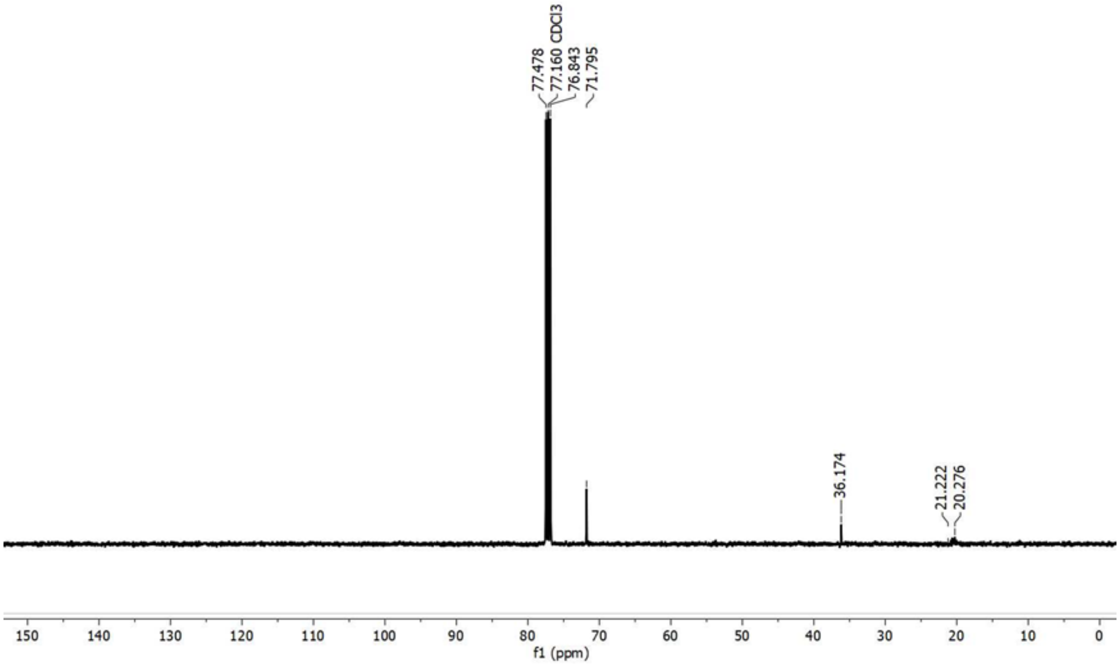

Figure S7. ^{13}C NMR spectrum of **NPG-d6** ($CDCl_3$, 400 MHz, 298 K)

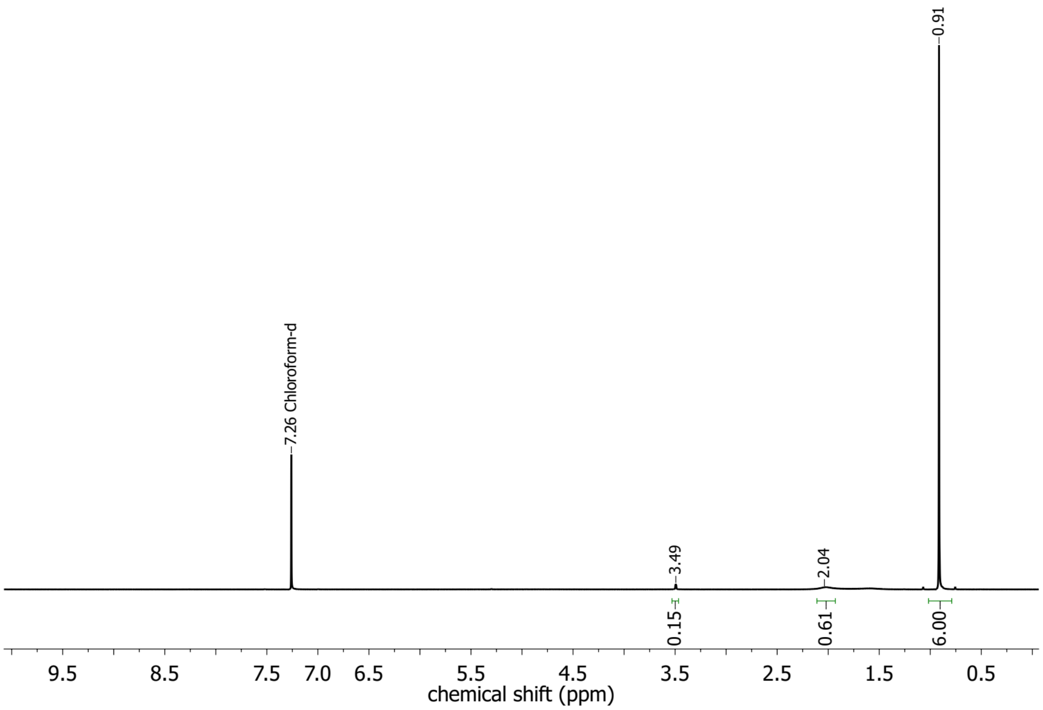

Figure S8. ^{1}H NMR spectrum of **NPG-d4+2** ($CDCl_3$, 400 MHz, 298 K)

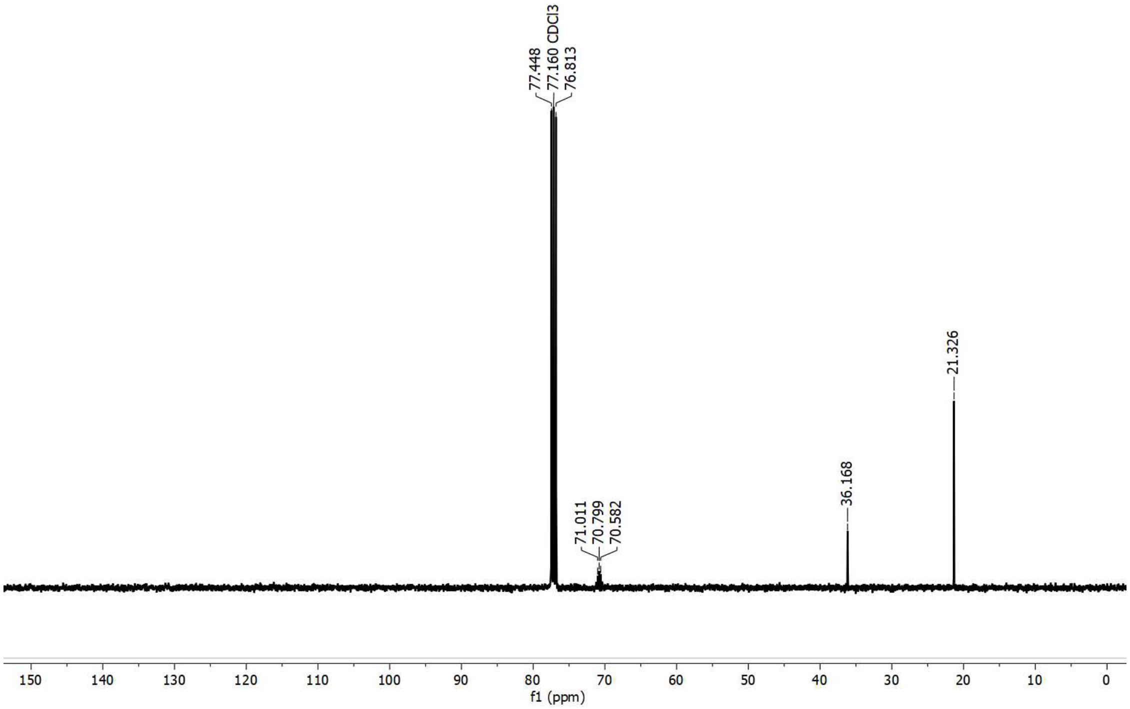

Figure S9. ^{13}C NMR spectrum of **NPG-d6** ($CDCl_3$, 400 MHz, 298 K)

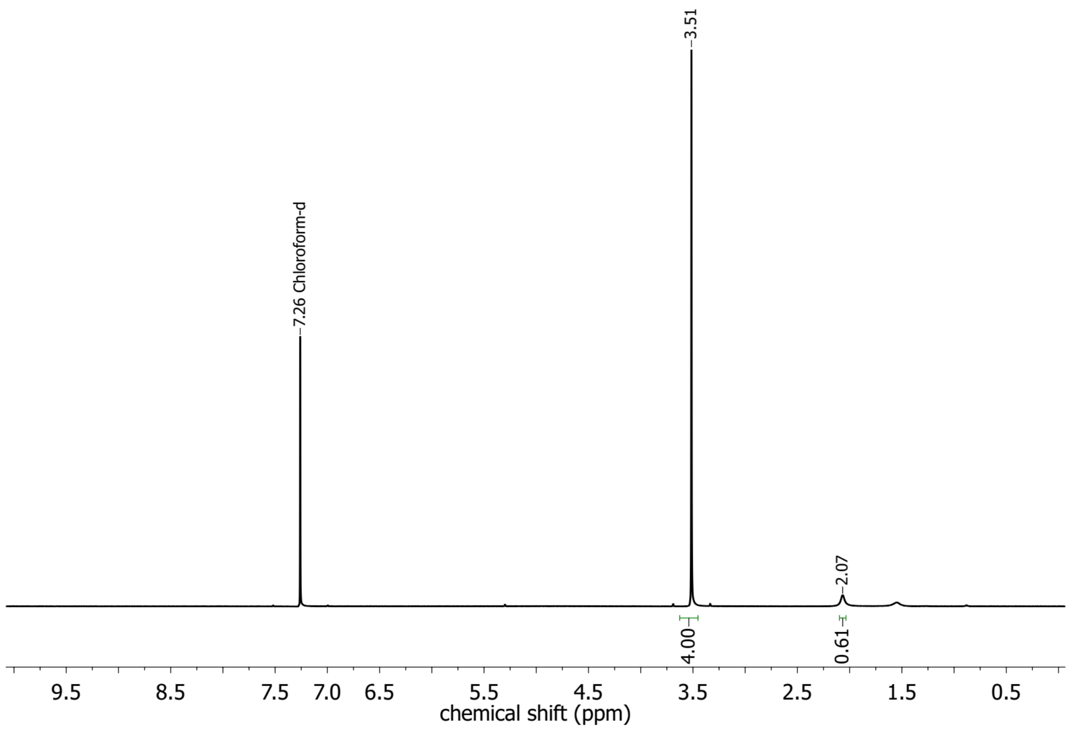

***Figure S*10.** ^{1}H NMR spectrum of **NPG-d6+2** ($CDCl_3$, 400 MHz, 298 K)

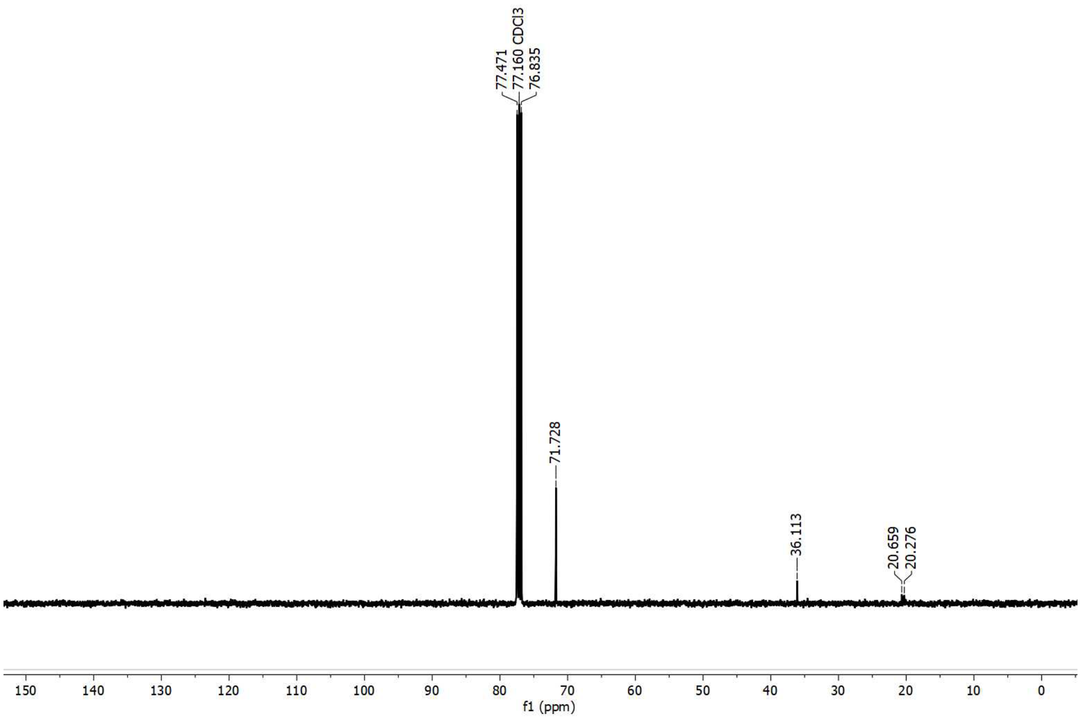

***Figure S*11.** ^{13}C NMR spectrum of **NPG-d6+2** ($CDCl_3$, 400 MHz, 298 K)

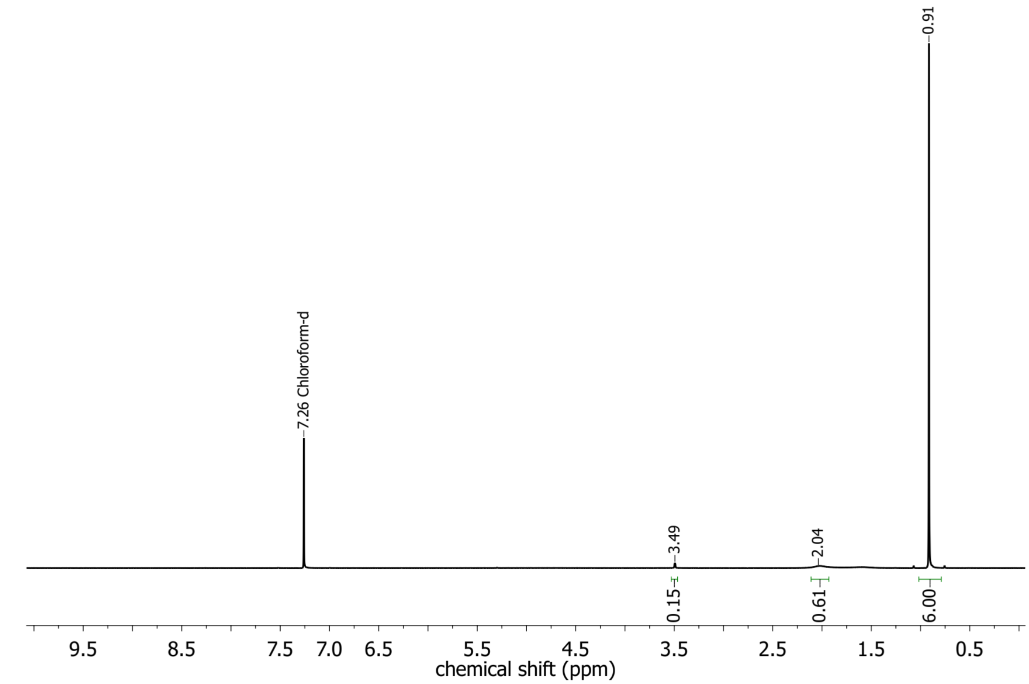

Figure S**12.** ^{1}H NMR spectrum of **NPG-d4+2** ($CDCl_3$, 400 MHz, 298 K)

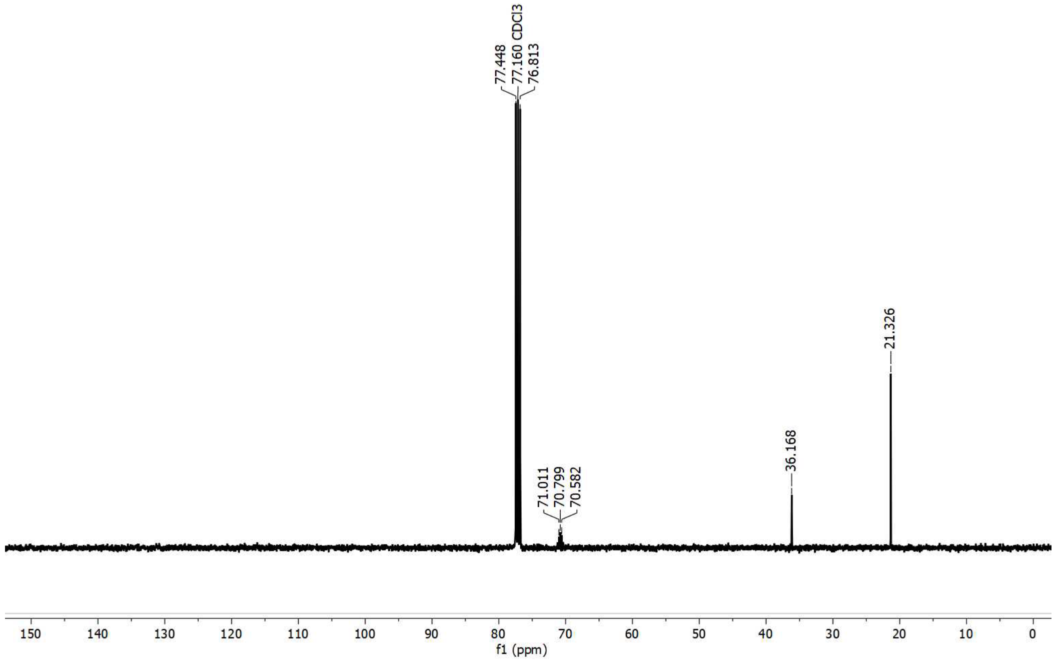

Figure S**13.** ^{13}C NMR spectrum of **NPG-d4+2** ($CDCl_3$, 400 MHz, 298 K)

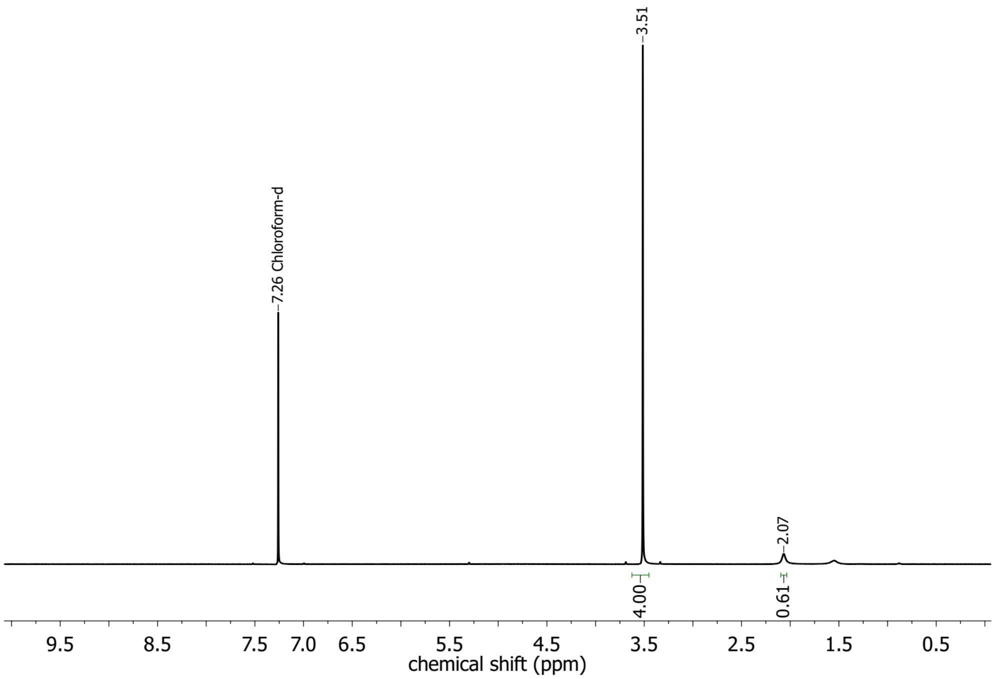

***Figure S*14.** ^{1}H NMR spectrum of **NPG-d6+2** ($CDCl_3$, 400 MHz, 298 K)

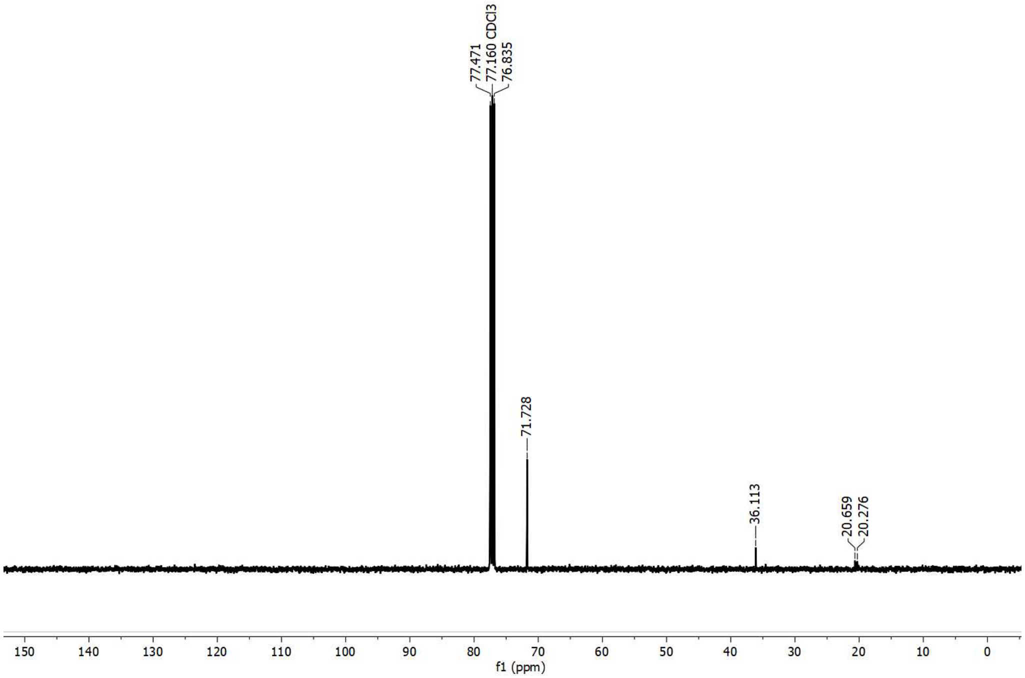

***Figure S*15.** ^{13}C NMR spectrum of **NPG-d6+2** ($CDCl_3$, 400 MHz, 298 K)

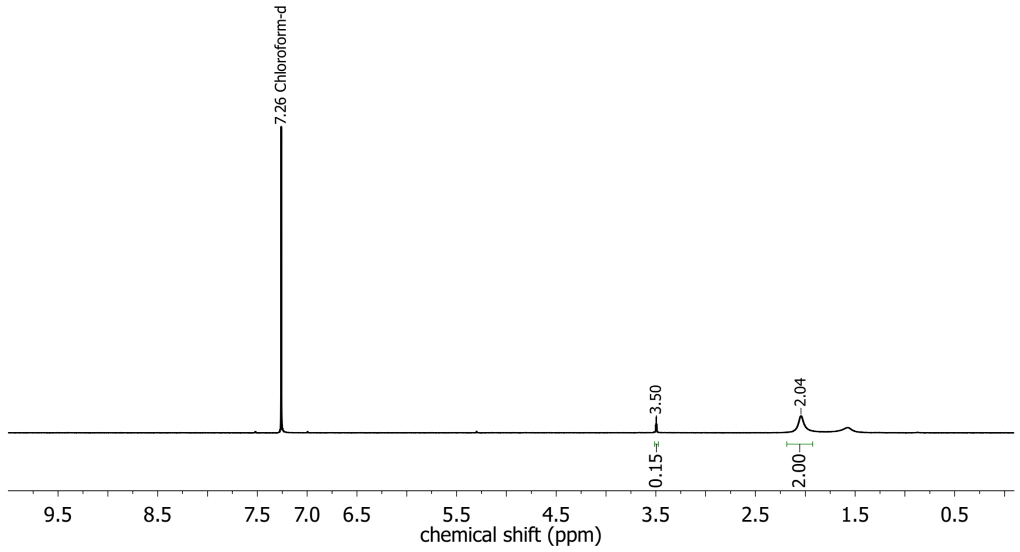

Figure S16. ^{1}H NMR spectrum of **NPG-d6+4** ($CDCl_3$, 400 MHz, 298 K)

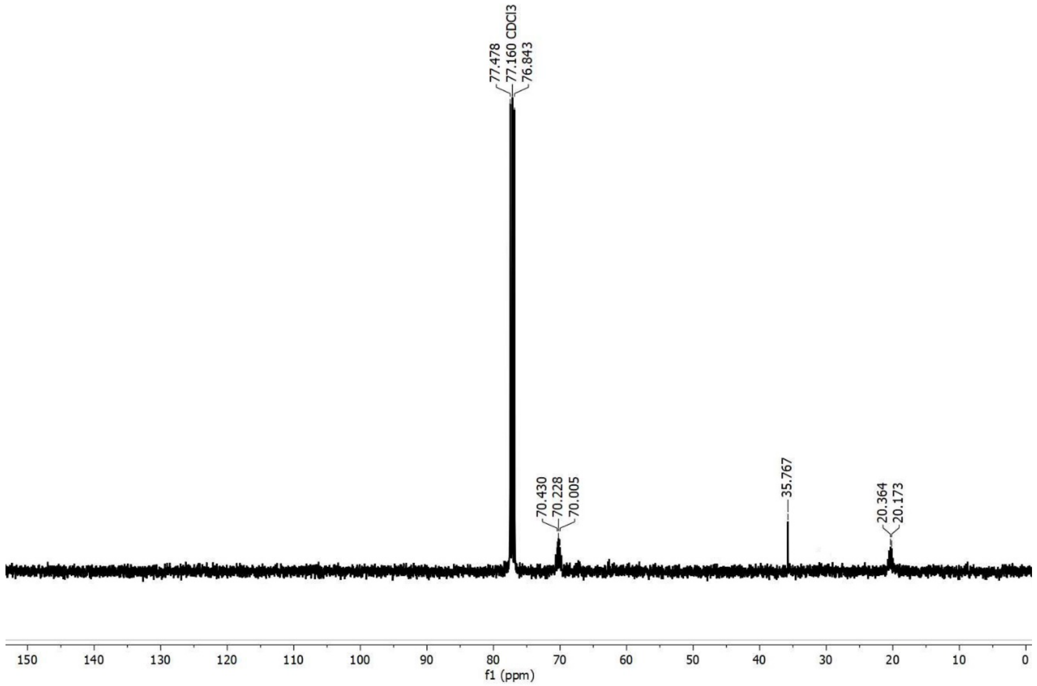

Figure S17. ^{13}C NMR spectrum of **NPG-d6+4** ($CDCl_3$, 400 MHz, 298 K)

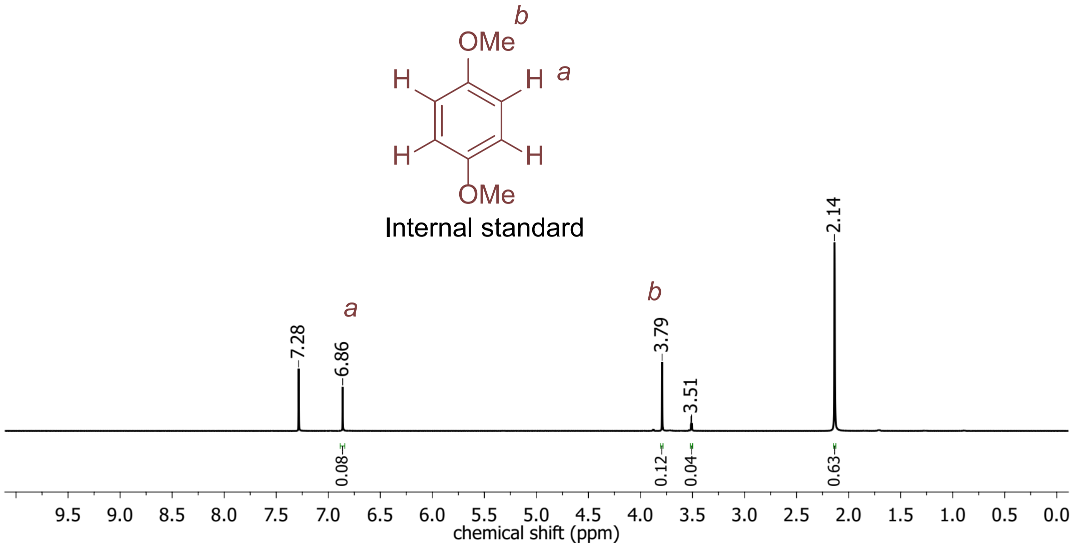

***Figure S*18.** ^{13}C NMR spectrum of **NPG-d6+4+2** ($CDCl_3$, 400 MHz, 298 K)

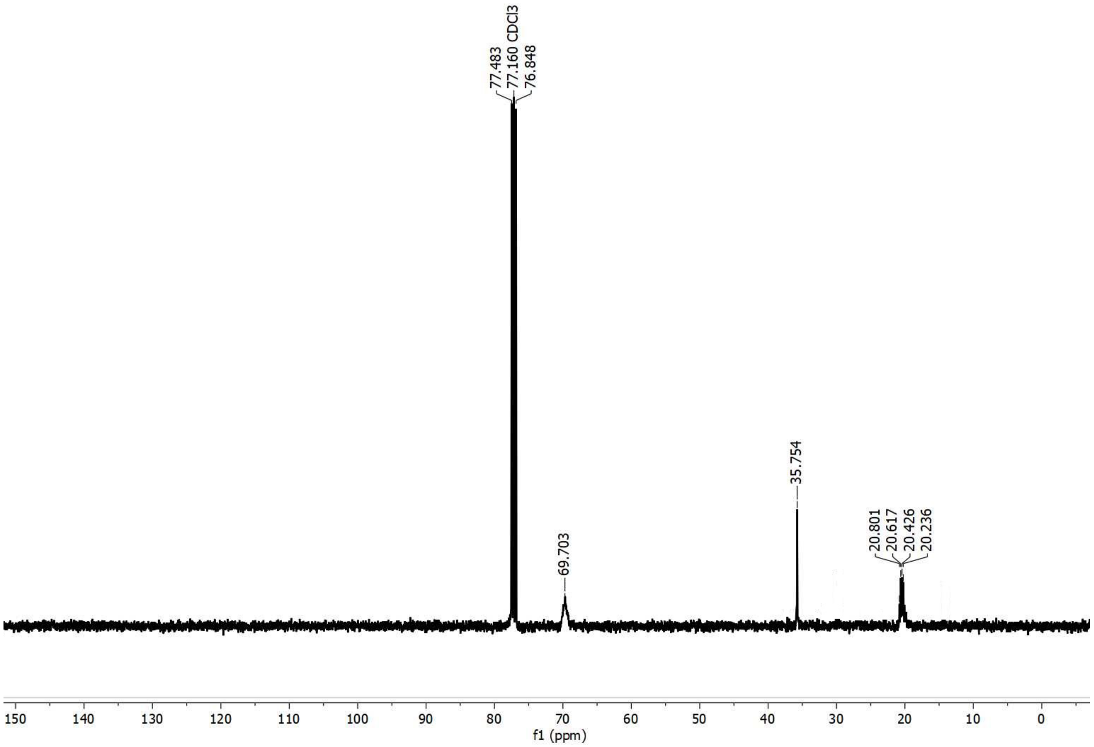

Figure S19. ^{13}C NMR spectrum of **NPG-d6+4+2** ($CDCl_3$, 400 MHz, 298 K)

5. Detection of Trace Impurity

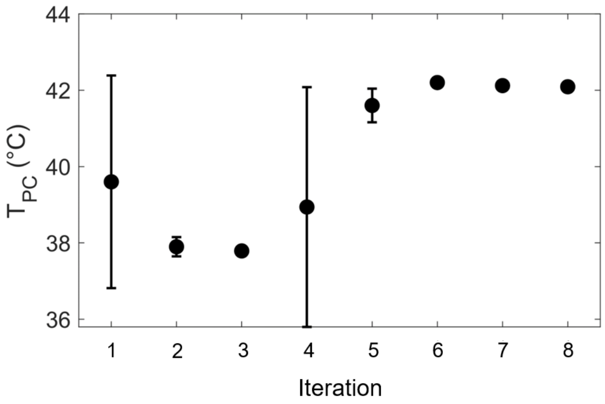

Figure S20. The onset temperature of the plastic crystal transition determined via a TA Q2000 DSC for NPG-d4 synthesized using the method outlined in the general synthetic procedure of Section 2, but under different purification methods. The purification methods for each iteration are as follows: Iterations 1 and 2 are controls in which no purification steps were taken, iteration 3 was recrystallized from solution, iteration 4 and 5 were solvent washed with DCM only, iteration 6 was solvent washed with DCM and n-pentane, and iteration 7 and 8 were sublimated and then washed with DCM and n-pentane. The error bars correspond to ±2σ of three independently prepared samples. Iteration 8 corresponds to the final dataset used in the main text.

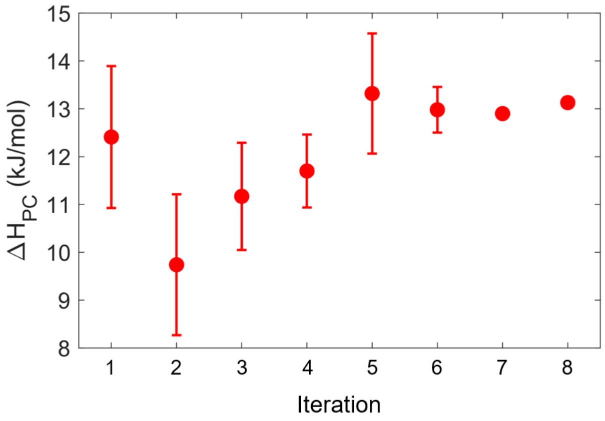

Figure S21. Molar enthalpy values determined via a TA Q2000 DSC for NPG-d4 prepared under different synthesis and purification. The purification methods for each iteration are as follows: Iterations 1 and 2 are controls in which no purification steps were taken, iteration 3 was recrystallized from solution, iteration 4 and 5 were solvent washed with DCM only, iteration 6 was solvent washed with DCM and n-pentane, and iteration 7 and 8 were sublimated and then washed with DCM and n-pentane. The error bars correspond to ±2σ of three independently prepared samples. For iterations 7 and 8, the error bar is smaller than the size of the marker. Iteration 8 corresponds to the final dataset used in the main text.

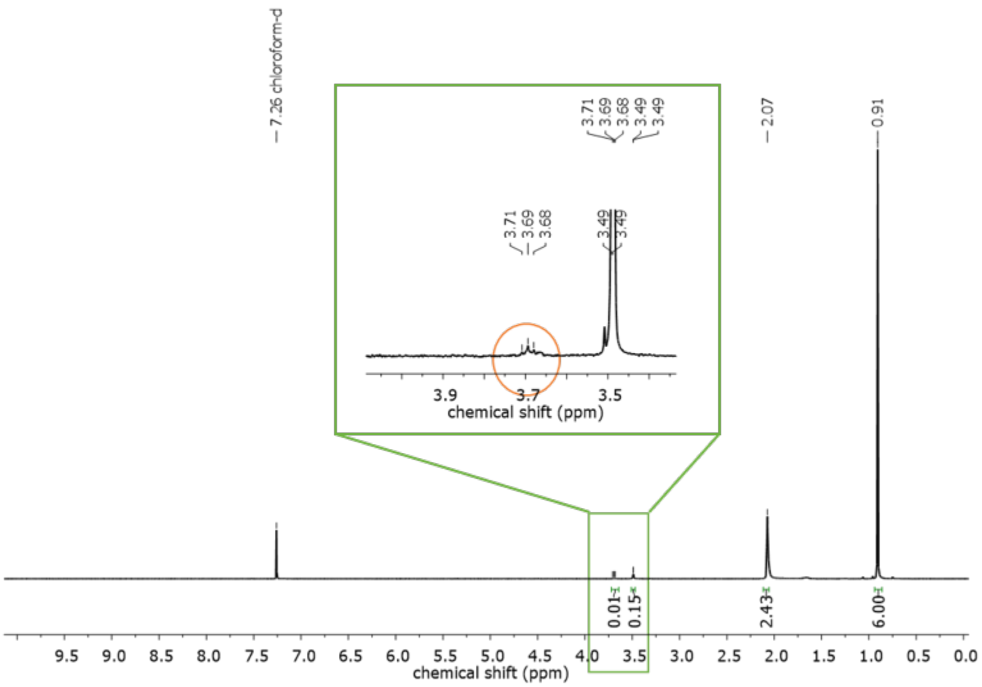

Figure S22. ^{1}H NMR spectrum of impure **NPG-d4** ($CDCl_3$, 400 MHz, 298 K). The boxed region (3.45-4.75 ppm) is expanded in the inset to show the trace impurity, which is circled in orange in the inset. This signal is absent from the ^{1}H NMR spectrum of NPG-d4 purified via sublimation and washing with DCM and n-pentane, as showing in *Figure S-3*.

6. Mass Spectra of Compounds

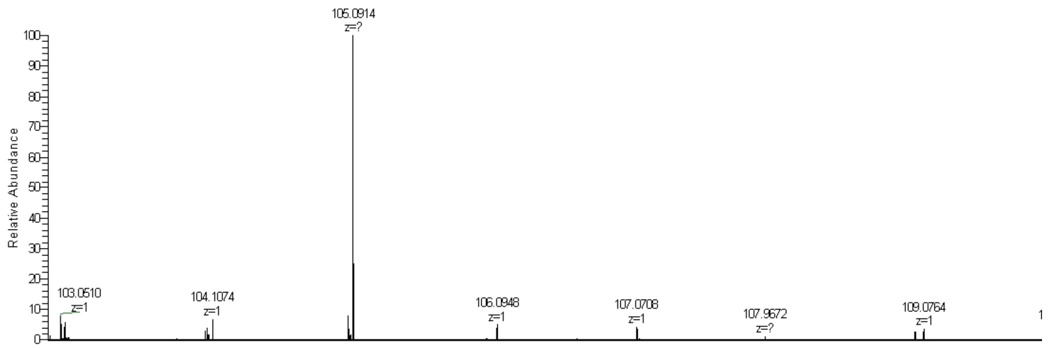

Figure S23. HRMS spectrum of **NPG-d2.** HRMS-ESI$^+$ (m/z): $[M+H]^+$ for $C_5H_{13}O_2^+$ calculated, 105.0910; found, 105.0914.

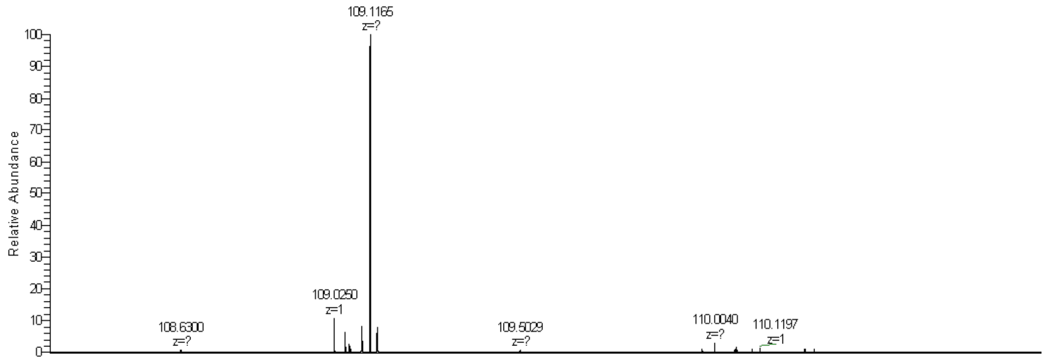

Figure S24. HRMS spectrum of **NPG-d4.** HRMS-ESI$^+$ (m/z): $[M+H]^+$ for $C_5H_9D_4O_2^+$ calculated, 109.1161; found, 109.1166.

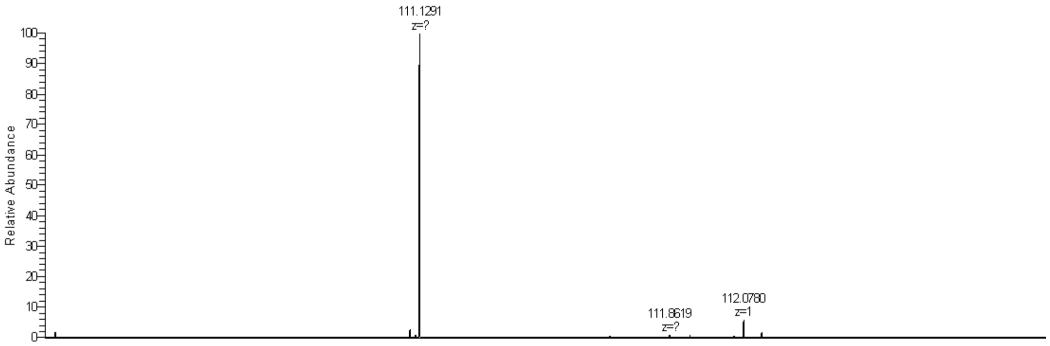

Figure S25. HRMS spectrum of **NPG-d6**. HRMS-ESI$^+$ (m/z): $[M+H]^+$ calcd. For $C_5H_7D_6O_2^+$, 111.1287; found, 111.1291.

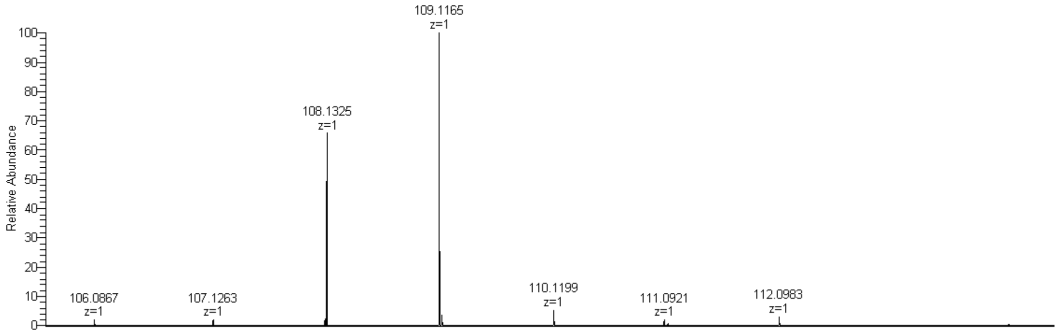

Figure S26. HRMS spectrum of **NPG-d4+2**. HRMS-ESI$^+$ (m/z): $[M+4]^+$ for $C_5H_9D_4O_2^+$ calculated, 109.1161; found, 109.1165.

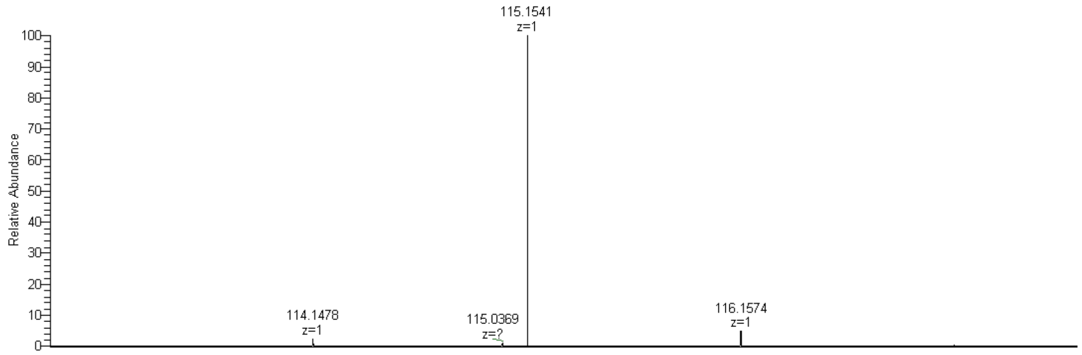

Figure S27. HRMS spectrum of **NPG-d6+4.** $[M+H]^+$ for $C_5H_3D_{10}O_2^+$ calculated, 115.1538; found, 115.1541

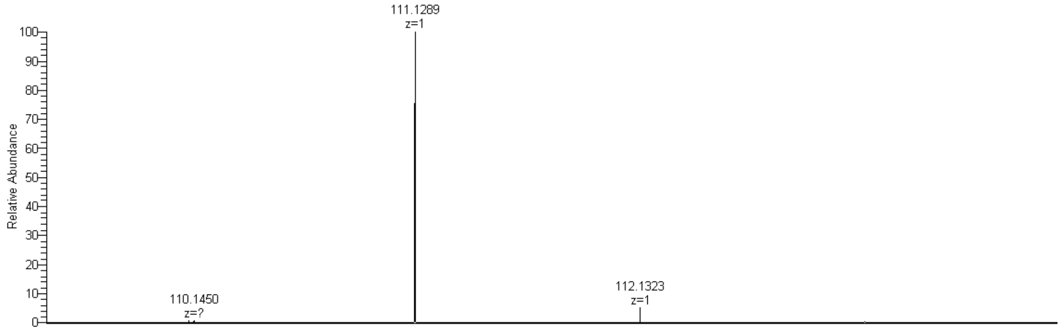

Figure S28. HRMS spectrum of **NPG-d6+2.** HRMS-ESI$^+$ (m/z): $[M+H]^+$ for $C_5H_7D_6O_2^+$ calculated, 111.1287; found, 111.1289.

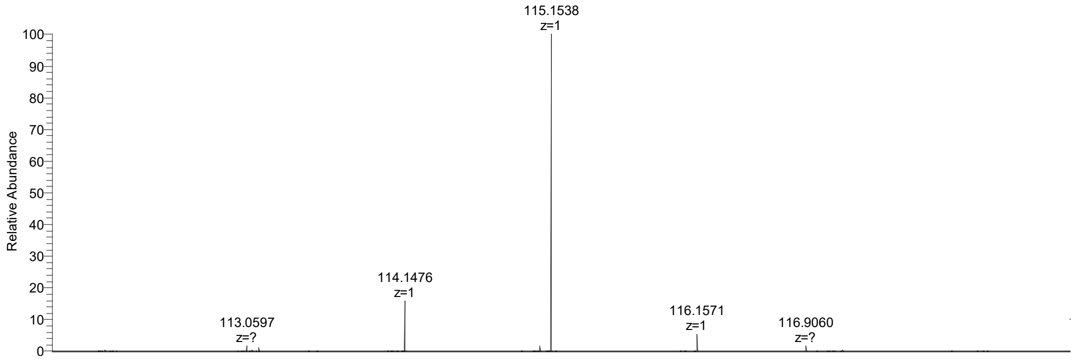

Figure S29. HRMS spectrum of **NPG-d6+4+2.** HRMS-ESI$^+$ (m/z): $[M+H]^+$ for $C_5H_3D_{10}O_2^+$ calculated, 115.1538; found, 115.1538.

7. Thermophysical Properties

7.1 Heat Capacity

Analysis of the heat capacity for pure NPG and comparison to literature for NPG-d2 and the fully deuterated analog NPG-d6+4+2 show that deuteration of the methyl group increases the molar heat capacity of both the ordered and plastic crystal phases. The discontinuity in heat capacity at 314.8 K in NPG, NPG-d2, and NPG-d4, and 311.8 K for NPG-d6+4+2 corresponds to the plastic crystal transition of each respective compound (Fig. 4). The increased magnitude of the heat capacity for both compounds past this transition reflects the rotationally disordered nature, i.e. the high frequency jumps between orientationally disordered states of the plastic crystal phase. The heat capacity for NPG-d6+4+2 recorded by Kamae et al.[1] appears to be of higher magnitude than NPG, and NPG-d2 in both ordered and plastic crystal phases. This could potentially be explained by the type of torsional motion occurring below the plastic crystal transition and the associated difference in vibrational frequency. The methyl groups of NPG are known to be thermally active above approximately 60-100 K,[2, 3] so substituting hydrogen with deuterium would alter the vibrational frequency (ν) due to the well-established relationship $\nu = \sqrt{k/\mu}$, where k is the force constant due to bonding and μ is the reduced mass. The lower vibrational frequency for the fully deuterated molecule suggests that more vibrational modes are activated at lower temperature, resulting in larger heat capacity magnitudes than partial or non-deuterated analogs. This hypothesis is supported by low-temperature data reported by Kamae *et al*., which shows the heat capacity values of NPG, NPG-d2, and fully deuterated NPG-d6+4+2 starting to converge as temperature decreases into the regime in which vibrational modes should no longer be thermally active (below 50 K).

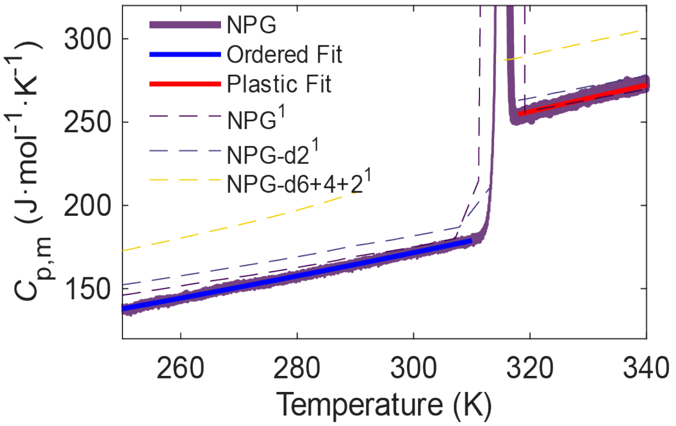

Figure S30. Molar heat capacity of NPG determined via a Setaram Microcalvet Calorimeter compared to data obtained by Kamae et al.[1] The relative uncertainty for the molar heat capacity in this temperature range is $u_\mathrm{r}(C_\mathrm{p,m}) = \pm 0.015$, and the experimental values exhibit a relative deviation of ±0.02 with respect to the referenced dataset.

To account for the difference in temperature of transition and compare the magnitude of enthalpy and entropy change at a reference transition temperature, the isobaric heat capacity and temperature of transition of NPG was used. The values of experimental heat capacity for the low and high temperature solid phases of NPG were determined via microcalorimetry and fitted

with a polynomial regression of the form $C_p(T) = a + b\left(\frac{T}{T_o}\right) + c\left(\frac{T}{T_o}\right)^2$, where T_o is the plastic crystal transition temperature, which is taken as 314.8 K for NPG.

Table S1: The calculated coefficients for the equation of the molar heat capacity in the ordered (250 to 310 K) and plastic (318 to 390 K) crystal phases.

	a	b	c
	J·mol^{-1}·K^{-1}	J·mol^{-1}·K^{-1}	J·mol^{-1}·K^{-1}
NPG $C_{p,m_{oc}}$	32.9892	65.3956	84.0281
NPG $C_{p,m_{dc}}$	-2.1252	256.8357	-2.5750

These equations were used to calculate the sensible heat associated with shifting the deuterated molecule's transitions to the reference temperature, as outlined in supplementary Section 7.3. The resultant corrections fall within the range of the experimental uncertainty and thus were not included in the main text.

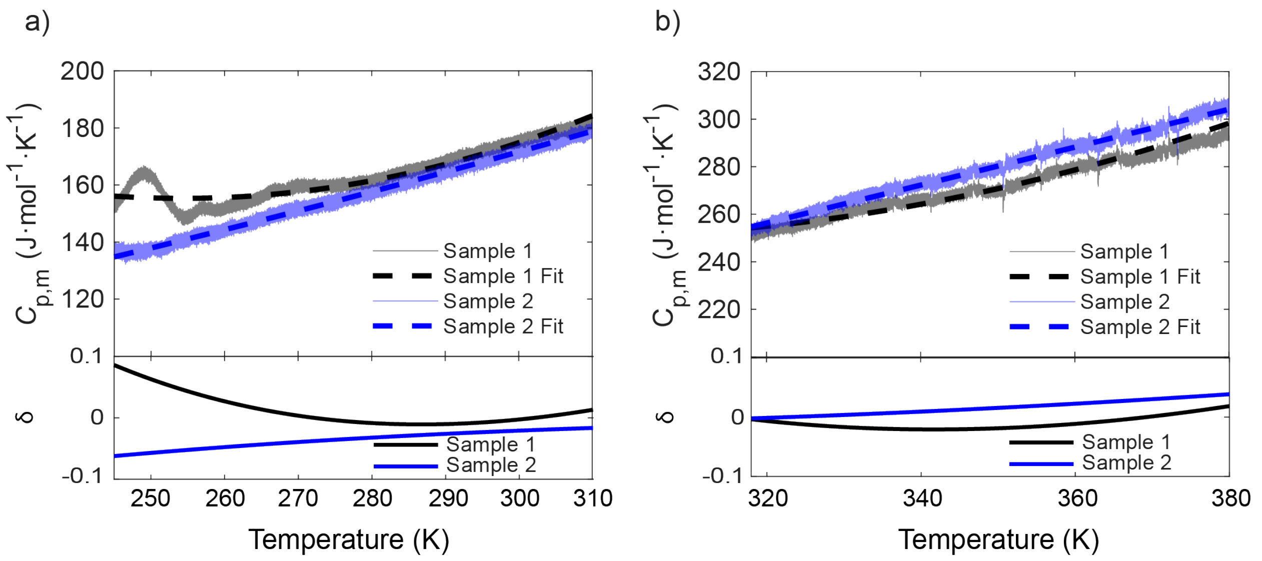

Figure S31: Raw and fitted molar heat capacities of a) the ordered crystal phase and b) plastic crystal phase of two samples of purified NPG. The lower part of figure a) and b) represent the deviation between each samples respective heat capacity as compared to the reference NPG data from Kamae et al.[1]

7.2 Thermophysical Properties of the Melt Transition

Table S2: Thermodynamic properties of the melt transition in deuterated NPG.

	T_{fus} [a]	ΔH_{fus} [b]	ΔH_{fus} [b]	ΔS_{fus} [b]
	K	J·g^{-1}	kJ·mol^{-1}	J·mol^{-1}·K^{-1}
NPG	402.6 ± 0.2	42.3 ± 1.5	4.4 ± 0.2	11.0 ± 0.4
NPG-d2	402.4 ± 0.2	41.6 ± 0.5	4.4 ± 0.1	11.0 ± 0.1
NPG-d4	401.4 ± 0.2	42.8 ± 0.1	4.6 ± 0.1	11.5 ± 0.1
NPG-d4+2	401.3 ± 0.2	42.0 ± 0.9	4.6 ± 0.1	11.5 ± 0.3
NPG-d6	401.6 ± 0.2	42.0 ± 2.5	4.6 ± 0.3	11.5 ± 0.7
NPG-d6+2	400.6 ± 0.2	39.5 ± 1.2	4.4 ± 0.2	11.0 ± 0.4
NPG-d6+4	400.2 ± 0.2	40.1 ± 0.7	4.6 ± 0.1	11.5 ± 0.2
NPG-d6+4+2	--	--	--	--

[a] The reported uncertainty is based on $u_r(T)$ = $\pm$ 0.0004. [b] $u_r(\Delta H_{\text{tr}})$ = $\pm$ 0.025. The reported uncertainty is ± 2σ of the number 3 individually prepared samples.

7.3 Thermophysical Properties of the Solid-Solid Plastic Crystal Transition, Adjusted to a Single Reference Temperature

Table S3: Corrected thermodynamic data of the plastic crystal transition

	T_{tr}	$\Delta H_{\text{tr,E}}$	$\Delta H_{\text{tr,C}}$	$\Delta S_{\text{tr,E}}$	$\Delta S_{\text{tr,C}}$
	K	kJ·mol^{-1}	kJ·mol^{-1}	J·mol^{-1}·K^{-1}	J·mol^{-1}·K^{-1}
NPG	314.8	12.4 ± 0.4	--	39.4 ± 1.4	--
NPG-d2	314.8	12.3 ± 0.2	--	39.2 ± 0.7	--
NPG-d4	315.3	12.9 ± 0.1	12.9	41.0 ± 0.3	41.0
NPG-d4+2	315.6	13.1 ± 0.3	13.1	41.6 ± 0.8	41.5
NPG-d6	312.2	12.5 ± 0.7	12.7	40.1 ± 2.2	40.4
NPG-d6+2	312.2	12.3 ± 0.4	12.5	39.4 ± 1.1	39.6
NPG-d6+4	312.8	12.7 ± 0.2	12.8	40.6 ± 0.6	40.7
NPG-d6+4+2	312.1	12.6 ± 2.0	12.8	40.3 ± 6.3	40.6

$\Delta H_{\text{tr,E}}$ and $\Delta S_{\text{tr,E}}$ characterizes the molar enthalpy and entropy of the plastic crystal transition determined directly from differential scanning calorimetry. $\Delta H_{\text{tr,C}}$ and $\Delta S_{\text{tr,C}}$ characterizes the corrected enthalpy and entropy calculated by adjusting the respective transition temperature (T') to pure NPG's ($T_{\text{ref}} = 314.8$ K) using the following equations:

$$\Delta H_{\text{tr},C} = \Delta H_{\text{tr},E} + \left[\int_{T'}^{T_{\text{ref}}} C_{p_{\text{dc}}} dT - \int_{T'}^{T_{\text{ref}}} C_{p_{\text{oc}}} dT\right] \quad \text{eq. S-1}$$

$$\Delta S_{\mathrm{tr,C}} = \frac{\Delta H_{\mathrm{tr,C}}}{T_{\mathrm{ref}}} \qquad \text{eq. S-2}$$

Where $C_{p_{\mathrm{dc}}}$ and $C_{p_{\mathrm{oc}}}$ are the isobaric heat capacities of the disordered and ordered crystal phase determined via a Setaram Microcalorimeter.

8. Conformational Analysis

8.1 Molecular Dynamics of Ordered Crystal Phase Conformations

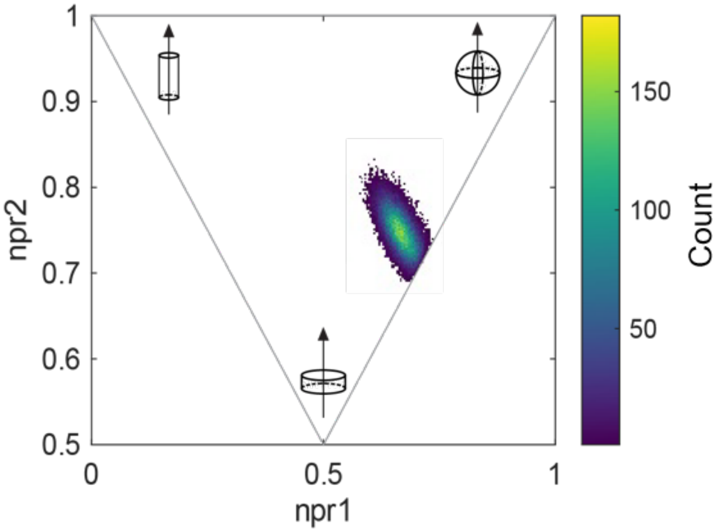

Figure S32: Density map of molecular conformations sampled from molecular dynamics simulations of the ordered crystal phase in normalized principal moment of inertia (npr) space. The color scale represents frequency of occurrence of conformations within each bin of the 2D histogram, with higher intensities corresponding to more frequently sampled geometries.

8.2 Molecular Dynamics of Liquid Phase Conformations

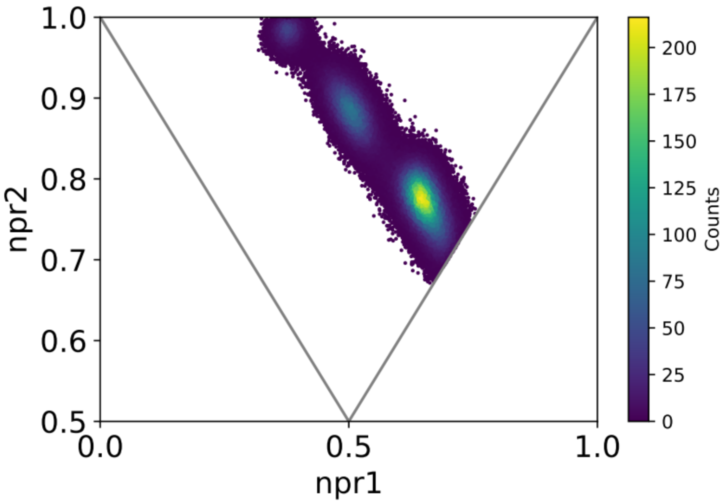

Figure S33: Density map of molecular conformations sampled from molecular dynamics simulations of the liquid phase of NPG in normalized principal moment of inertia (npr) space. The color scale represents frequency of occurrence of conformations within each bin of the 2D histogram, with higher intensities corresponding to more frequently sampled geometries.

8.3 Planar Representation of Normalized Principal Moments

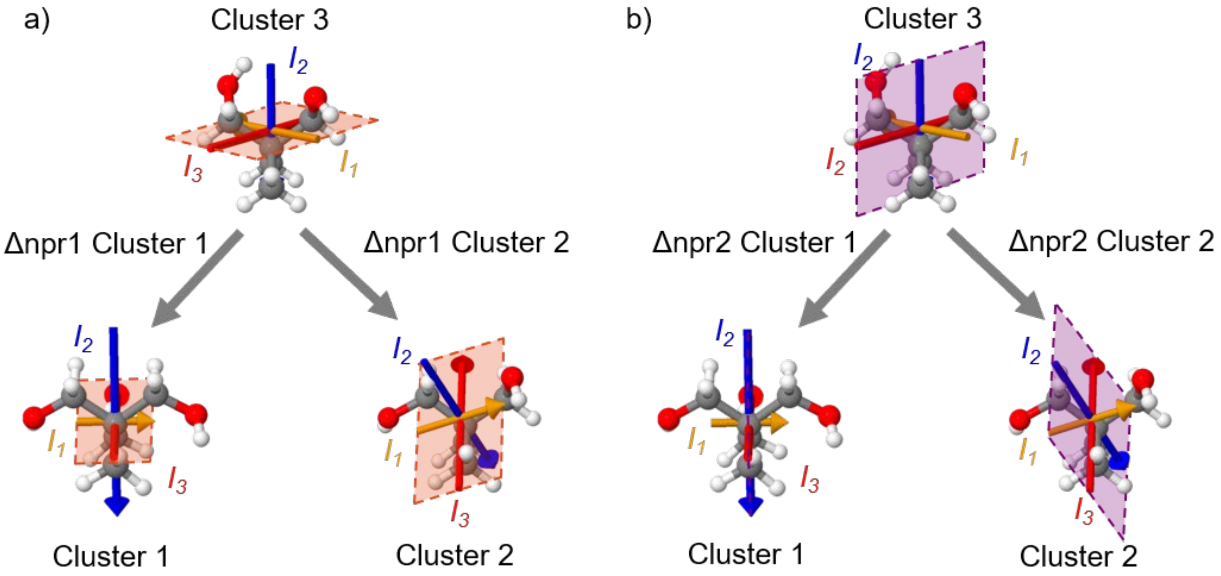

Figure S34: Planar representation of a) the first normalized principal moment of inertia (npr1) and b) the second normalized principal moment of inertia (npr2). It can be observed that the methyl group primarily impacts the vector npr1, as the planar representation of npr2 roughly bisects the methyl group in each cluster.

9. Sphericity Analysis

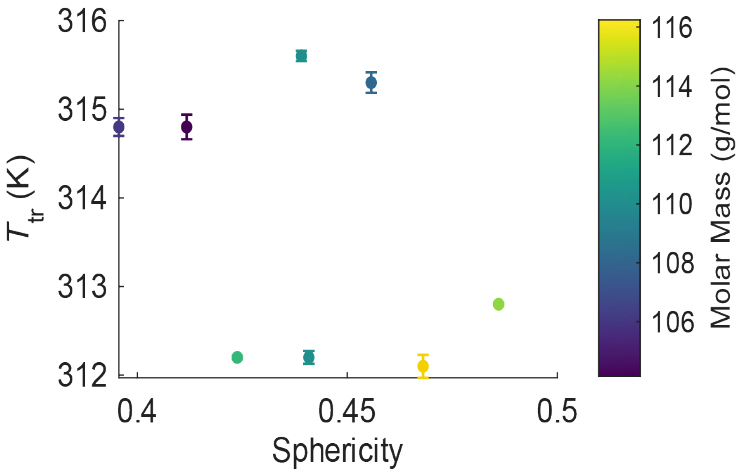

Figure S35: The temperature of the plastic crystal transition (T_{tr}) dependence on the sphericity of the molecules in the ordered crystal phase, calculated as $\mathrm{S} = \mathrm{npr1} + \mathrm{npr2} - 1$. The error bars correspond to ±2σ of *n* independently prepared samples, where *n* = 2 for NPG-d6+4+2, and 3 for all other compounds.

10. References

1. Kamae, R.; Suenaga, K.; Matsuo, T.; Suga, H., Low-temperature thermal properties of 2,2-dimethyl-1,3-propanediol and its deuterated analogues. *The Journal of Chemical Thermodynamics* **2001,** *33* (5), 471-484.
2. Li, B.; Kawakita, Y.; Ohira-Kawamura, S.; Sugahara, T.; Wang, H.; Wang, J.; Chen, Y.; Kawaguchi, S. I.; Kawaguchi, S.; Ohara, K.; Li, K.; Yu, D.; Mole, R.; Hattori, T.; Kikuchi, T.; Yano, S.-i.; Zhang, Z.; Zhang, Z.; Ren, W.; Lin, S.; Sakata, O.; Nakajima, K.; Zhang, Z., Colossal barocaloric effects in plastic crystals. *Nature* **2019,** *567* (7749), 506-510.
3. Rendell-Bhatti, F.; Appel, M.; Inglis, C. S.; Dilshad, M.; Mehta, N.; Radcliffe, J.; Moya, X.; MacLaren, D. A.; Boldrin, D., Direct Observation of Thermal Hysteresis in the Molecular Dynamics of Barocaloric Neopentyl Glycol. *ACS Applied Energy Materials* **2025,** *8* (7), 4793-4802.
